# First survey and functional annotation of prohormone and convertase genes in the pig

**DOI:** 10.1186/1471-2164-13-582

**Published:** 2012-11-15

**Authors:** Kenneth I Porter, Bruce R Southey, Jonathan V Sweedler, Sandra L Rodriguez-Zas

**Affiliations:** 1 Department of Animal Sciences, University of Illinois, 1207 W Gregory Dr, Urbana, IL, 61801, USA; 2 Department of Chemistry, University of Illinois, Urbana, IL, USA

**Keywords:** Prohormone, Prohormone convertase, Neuropeptide, Pig genome, Gene expression profile, Cleavage

## Abstract

**Background:**

The pig is a biomedical model to study human and livestock traits. Many of these traits are controlled by neuropeptides that result from the cleavage of prohormones by prohormone convertases. Only 45 prohormones have been confirmed in the pig. Sequence homology can be ineffective to annotate prohormone genes in sequenced species like the pig due to the multifactorial nature of the prohormone processing. The goal of this study is to undertake the first complete survey of prohormone and prohormone convertases genes in the pig genome. These genes were functionally annotated based on 35 gene expression microarray experiments. The cleavage sites of prohormone sequences into potentially active neuropeptides were predicted.

**Results:**

We identified 95 unique prohormone genes, 2 alternative calcitonin-related sequences, 8 prohormone convertases and 1 cleavage facilitator in the pig genome 10.2 assembly and trace archives. Of these, 11 pig prohormone genes have not been reported in the UniProt, UniGene or Gene databases. These genes are *intermedin*, *cortistatin*, *insulin-like 5*, *orexigenic neuropeptide QRFP*, *prokineticin 2*, *prolactin-releasing peptide*, *parathyroid hormone 2*, *urocortin*, *urocortin 2*, *urocortin 3*, and *urotensin 2-related peptide*. In addition, a novel *neuropeptide S* was identified in the pig genome correcting the previously reported pig sequence that is identical to the rabbit sequence. Most differentially expressed prohormone genes were under-expressed in pigs experiencing immune challenge relative to the un-challenged controls, in non-pregnant relative to pregnant sows, in old relative to young embryos, and in non-neural relative to neural tissues. The cleavage prediction based on human sequences had the best performance with a correct classification rate of cleaved and non-cleaved sites of 92% suggesting that the processing of prohormones in pigs is similar to humans. The cleavage prediction models did not find conclusive evidence supporting the production of the bioactive neuropeptides *urocortin 2*, *urocortin 3*, *torsin family 2 member A*, *tachykinin 4*, *islet amyloid polypeptide*, and *calcitonin receptor-stimulating peptide* 2 in the pig.

**Conclusions:**

The present genomic and functional characterization supports the use of the pig as an effective animal model to gain a deeper understanding of prohormones, prohormone convertases and neuropeptides in biomedical and agricultural research.

## Background

In addition to the importance in livestock production, the pig is a well-established biomedical model to study human health due to the genomic, anatomic and physiologic similarities with humans. A wide variety of human health traits including cancer, reproductive health, drug metabolism, wound healing, and cardiovascular disease have been successfully studied using the pig [1-4]. Underlying these and other important traits such as growth and development, feeding, stress, memory and susceptibility to substances abuse are neuropeptides, a class of cell-cell signaling peptides that have paracrine, endocrine, and autocrine effects [5,6]. Research in selected pig neuropeptides has offered insights into cell transplantation, nervous system diseases, and injury [7]. For example, Yang et al. [8] reported on the inhibitory effect of *neuromedin S* (*NMS*) on luteinizing hormone secretion which is mediated via *melanocyte-stimulating hormone* (*MSH*) neurons in the hypothalamus-pituitary axis of ovariectomized pigs. Kaminski et al. [9] concluded that hypothalamic peptides, *orexin A* and *orexin B*, were involved in the control of food intake, sleep patterns, autonomic and neuroendocrine systems, and may also affect reproductive functions through the influence on the hypothalamic-pituitary-ovarian axis in pigs.

The identification of neuropeptides is more difficult than most proteins because neuropeptides are derived from larger prohormone proteins as a result of complex enzymatic processing. The conversion of the large prohormone to one or multiple smaller neuropeptides involves cleavage by multiple prohormone convertases and additional post-translational modifications such as amidation and glycosylation [5]. This complex processing of prohormones into neuropeptides challenges the identification of neuropeptide genes across genomes solely based on sequence homology to better understood species [5,6,10].

Only 40 prohormone and 2 prohormone convertase genes have been empirically confirmed in the pig compared to approximately 100 genes identified in human, rat, mouse, cow and rhesus monkey [11-13]. This partial knowledge of the prohormone, prohormone convertase and associated neuropeptides in the pig is a critical shortcoming, especially considering the important role of pig in biomedical and agricultural research [1]. In addition, few gene expression studies have discussed the expression profile of pig prohormone or prohormone convertase genes. Ross et al. [14] found that estradiol treatment at day 9 of gestation was associated with changes in the expression of the prohormone *neuromedin* (*NMB*) in the endometrium of glits. Hausman et al. [15] concluded that the expression of *neuropeptide Y* (*NPY*) was down-regulated with age in gilts ranging from 90 to 210 days old.

Understanding the role of neuropeptides in human and livestock traits using the pig as biomedical model requires a comprehensive knowledge of the neuropeptide complement in the recently released pig genome (SScrofa 10.2v18, [16]). This understanding includes the identification of prohormone and prohormone convertase genes, prediction of cleavage sites in prohormones that may result in potentially bioactive neuropeptides, and characterization of gene expression and protein abundance across conditions to gain insights into the role of neuropeptides. A complete survey of the prohormone and prohormone convertase genes in the pig supports the interpretation of gene expression experiments and improves the effectiveness of tandem mass spectrometry studies to identify neuropeptides [17-19]. Functional annotation of these genes can be obtained by the analysis of the large number of gene expression experiments already available [20,21]. To address the lack of a comprehensive understanding of the prohormone and prohormone convertase genes in the pig, we present the first comprehensive survey and functional annotation of these genes. An all-inclusive catalogue of prohormone and prohormone convertase genes known in other species was used to search complementary pig genome databases. These genes were then characterized by analyzing a large number of gene expression experiments across a wide range of conditions. The potential cleavage sites of prohormones that can result in bioactive neuropeptides were predicted and compared to the cleavages based on known neuropeptide sequences.

## Results and discussion

### Pig prohormone genes

A comprehensive catalogue of 95 potential pig prohormone genes, 8 prohormone convertase genes and 1 prohormone convertase facilitator gene (*7B2*) were identified in the pig genome. Table 1 lists the genes and the corresponding BLAST matches on the pig Genome, UniProt, Gene and UniGene databases. There are 11 previously unreported (i.e. without empirical confirmation) prohormone genes in the pig and complete sequences where only partially or incomplete sequences have been previously reported. Newly identified genes are *intermedin* (*ADM2*), *cortistatin* (*CORT*), *insulin-like 5* (*INSL5*), *orexigenic neuropeptide QRFP* (*OX26*), *prokineticin 2* (*PROK2*), *prolactin-releasing peptide* (*PRRP*), *parathyroid hormone 2* (*TIP39*), *urocortin* (*UCN1*), *urocortin 2* (*UCN2*), *urocortin 3* (*UCN3*), and *urotensin 2-related peptide* (*UTS2B*). This search also identified two different calcitonin protein entries in public databases that are isoforms of other calcitonin genes. Additional information on the comprehensive catalogue of genes is available [see Additional file 1]. The predicted prohormone protein sequences with cleavage sites identification is provided in the NeuroPred format [see Additional file 2].

**Table 1:** Prohormone and convertase genes identified across pig genome resources

**Type**^**a**^	**Symbol**	**Gene name**	**Genome sequence**^**b**^	**UniGene**^**c**^	**UniProt**^**d**^	**Gene**^**e**^
P	*ADM2*	*Intermedin*	complete	Not Found	F1RXU1	100517471
P	*ADM5*	*Adrenomedullin-5*	complete	Ssc.26627	A5LHG2	100101476
P	*ADML*	*Adrenomedullin*	complete	Ssc.314	P53366	397195
P	*ANF*	*Atrial natriuretic factor*	complete	Ssc.16245	P24259	397496
P	*ANFB*	*Natriuretic peptide B*	complete	Ssc.629	P07634	396844
P	*ANFC*	*C-type natriuretic peptide*	complete	Ssc.23867	P18104	493772
P	*APEL*	*Apelin*	complete	CU928865	Not Found	100625006
P	*AUGN*	*Augurin*	complete	Ssc.22487	F1SU23	100512958
P	*CALC*	*Calcitonin/calcitonin gene-related peptide 1*	complete	Ssc.14052	A6P7L6	100125547
P	*CALCalt*	*Preprocalcitonin gene-related peptide*	complete	Ssc.56129	A6P7L7	100124407
P	*CART*	*Cocaine- and amphetamine-regulated transcript protein*	complete	Ssc.15900	Q307W6	397252
P	*CCKN*	*Cholecystokinin*	complete	Ssc.717	P01356	397468
P	*CMGA*	*Chromogranin-A*	complete	Ssc.4653	P04404	397540
P	*COLI*	*Pro-opiomelanocortin*	complete	Ssc.14556	P01192	396863
P	*CORT*	*Cortistatin*	complete	Not Found	F1RIF7	100526112
P	*CRF*	*Corticoliberin*	complete	Ssc.69887	P06296	100127468
P	*CRSP1*	*Calcitonin receptor-stimulating peptide 1*	complete	Ssc.3741	Q862B1	396563
P	*CRSP2*	*Calcitonin receptor-stimulating peptide 2*	complete	Ssc.18558	Q766Y7	396574
P	*CRSP3*	*Calcitonin receptor-stimulating peptide 3*	complete	Ssc.17879	Q766Y6	396573
P	*CRSP3alt*	*Calcitonin-2*	complete	Not Found	A0A761	Not Found
P	*EDN1*	*Endothelin-1*	complete	Ssc.9364	P09558	396915
P	*EDN2*	*Endothelin-2*	complete	Not Found	Not Found	Not Found
P	*EDN3*	*Endothelin-3*	complete	Ssc.31972	A5A752	100049663
P	*GALA*	*Galanin*	complete	Ssc.713	P07480	397465
P	*GALP*	*Galanin-like peptide*	complete	Ssc.4875	Q9TT95	396772
P	*GAST*	*Gastrin*	complete	Ssc.644	P01351	445524
P	*GHRL*	*Obestatin*	complete	Ssc.440	Q9GKY5	396728
P	*GIP*	*Gastric inhibitory polypeptide*	complete	Ssc.38713	P01281	100621117
P	*GLUC*	*Glucagon*	complete	Ssc.17225	P01274	397595
P	*GON1*	*Progonadoliberin-1*	complete	Ssc.16310	P49921	397516
P	*GON2*	*Progonadoliberin-2*	Not Found	Not Found	F1S8B1	100523475
P	*GRP*	*Gastrin-releasing peptide*	complete	Ssc.13923	P63153	Not Found
P	*HEPC*	*Hepcidin*	complete	Ssc.376	Q8MJ80	397207
P	*IAPP*	*Islet amyloid polypeptide*	complete	Ssc.8324	Q29119	100520838
P	*IGF1*	*Insulin-like growth factor 1*	complete	Ssc.16231	P16545	397491
P	*IGF2*	*Insulin-like growth factor 2*	fragment	Ssc.9365	P23695	396916
P	*INS*	*Insulin*	complete	Ssc.583	P01315	397415
P	*INSL3*	*Insulin-like 3*	complete	Ssc.11990	P51461	397024
P	*INSL5*	*Insulin-like 5*	complete	Not Found	Not Found	100620109
P	*INSL6*	*Insulin-like 6*	complete	Ssc.46919	F1SK47	100158105
P	*KISS1*	*Metastasis-suppressor KiSS-1*	complete	Ssc.73565	B5M447	100145896
P	*MCH*	*Pro-melanin-concentrating hormone*	complete	Ssc.3287	Q9TTS8	396962
P	*MOTI*	*Motilin*	complete	Ssc.714	P01307	397466
P	*NEU1*	*Oxytocin*	complete	Ssc.15668	P01177	100152272
P	*NEU2*	*Neurophysin-2*	complete	Ssc.4210	P01183	396995
P	*NEUT*	*Neurotensin*	complete	Ssc.38680	F1SPX3	100739079
P	*NMB*	*Neuromedin-B*	complete	Ssc.2083	B0LUW4	100141313
P	*NMS*	*Neuromedin-S*	complete	Ssc.12508	C3UZJ1	100294685
P	*NMU*	*Neuromedin-U*	complete	Ssc.12508	P34964	100523263
P	*NPB*	*Neuropeptide B*	complete	Ssc.82498	Not Found	Not Found
P	*NPFF*	*Neuropeptide FF*	complete	Ssc.44958	F1SFP1	100518250
P	*NPS*	*Neuropeptide S*	complete	Ssc.73596	F1RSG4	100188981
P	*NPW*	*Neuropeptide W*	complete	Ssc.15796	Q8MI35	396680
P	*NPY*	*Neuropeptide Y*	complete	Ssc.15981	P01304	397304
P	*OREX*	*Orexin*	complete	Ssc.15983	O77668	397305
P	*OSTN*	*Osteocrin (Musclin)*	complete	Ssc.5148	A5JHN9	100049691
P	*OX26*	*Orexigenic neuropeptide QRFP*	complete	Not Found	F1S0X5	100524361
P	*PACA*	*Pituitary adenylate cyclase-activating polypeptide*	complete	Ssc.27598	P41535	414283
P	*PAHO*	*Pancreatic polypeptide*	complete	Ssc.456	P01300	397272
P	*PCSK1N*	*Proprotein convertase subtilisin/kexin type 1 inhibitor*	complete	Ssc.17429	Not Found	100621697
P	*PDGFA*	*Platelet-derived growth factor alpha polypeptide*	complete	Ssc.6173	F1RIZ0	100519764
P	*PDGFB*	*Platelet-derived growth factor beta polypeptide*	complete	Ssc.54182	P20034	100126843
P	*PDGFD*	*Platelet-derived growth factor D*	complete	Ssc.49835	F1SV50	100524161
P	*PDYN*	*Proenkephalin-B*	complete	Ssc.121	P01214	445529
P	*PENK*	*Proenkephalin*	complete	Ssc.11281	Q7M3H2/Q7M2Z7	100152093
P	*PNOC*	*Prepronociceptin*	complete	Ssc.15910	P55791	397257
P	*PROK2*	*Prokineticin 2*	fragment	EW633867	Not Found	100526076
P	*PRRP*	*Prolactin-releasing peptide*	fragment	Not Found	Not Found	Not Found
P	*PTHR*	*Parathyroid hormone-related peptide*	complete	Ssc.9991	Q866H2	396951
P	*PTHY*	*Parathyroid hormone*	complete	Ssc.668	P01269	399502
P	*PYY*	*Peptide YY*	complete	Ssc.63650	P68005	445018
P	*REL1*	*Pro-relaxin 1*	complete	Ssc.162	P01348	396891
P	*REL3*	*Relaxin 3*	complete	Ssc.42647	Q8HY17	503836
P	*RES18*	*Regulated endocrine-specific protein 18*	complete	Ssc.49266	F1SR77	100154377
P	*RFRP*	*Neuropeptide VF precursor*	complete	Ssc.75350	C4P9W1	100302024
P	*SCG1*	*Secretogranin-1*	complete	Ssc.15718	Q9GLG4	397154
P	*SCG2*	*Secretogranin-2*	complete	Ssc.13645	Q5FZP5	497237
P	*SCG3*	*Secretogranin-3*	complete	Ssc.6770	F1RYP7	100154760
P	*SECR*	*Secretin*	complete	Ssc.710	P63298	397464
P	*SLIB*	*Somatoliberin*	complete	Ssc.71374	P01287	100499556
P	*SMS*	*Somatostatin*	complete	Ssc.19520	P01168	494469
P	*SPXN*	*Spexin*	complete	Ssc.57764	F1SR03	100155886
P	*TIP39*	*Parathyroid hormone 2*	complete	Not Found	F1RHZ	100515141
P	*TKN1*	*Tachykinin, precursor 1*	complete	Ssc.18075	F1SF85	100525179
P	*TKN4*	*Tachykinin-4*	complete	Ssc.23153	F1RTB7	100511101
P	*TKNK*	*Tachykinin 3*	complete	Ssc.19565	P67934	492314
P	*TOR2X*	*Torsin family 2, member A*	fragment	Ssc.67158	B6VD08	100519815
P	*TRH*	*Prothyroliberin*	complete	Not Found	P62968	100513309
P	*UCN1*	*Urocortin*	Traces	Not Found	F8R6K7	Not Found
P	*UCN2*	*Urocortin 2*	complete	Not Found	F1SKM2	100521865
P	*UCN3*	*Urocortin 3*	complete	Not Found	F1RYW0	100737810
P	*UTS2*	*Urotensin 2*	complete	Ssc.437	Q95J46	397268
P	*UTS2B*	*Urotensin II-related peptide*	complete	Not Found	F1SFH3	100626084
P	*VEGFC*	*Vascular endothelial growth factor C*	complete	Ssc.12790	F1RT19	100525960
P	*VEGFD*	*Vascular endothelial growth factor D*	complete	Ssc.29289	F1SQU4	100155670
P	*VGF*	*Neurosecretory protein VGF*	fragment	Ssc.90772	Not Found	100624333
P	*VIP*	*Vasoactive intestinal peptide*	complete	Ssc.47759	E0Y441	100500718
C	*7B2*	*Neuroendocrine protein 7B2 (secretogranin 5)*	complete	Ssc.155	P01165	397110
C	*FURIN*	*Furin*	complete	Ssc.94009	F1RMJ1	100156882
C	*PCSK1*	*Proprotein convertase subtilisin/kexin type 1 PC1/3*	complete	Ssc.92884	Q28959	397103
C	*PCSK2*	*Proprotein convertase subtilisin/kexin type 2*	complete	Ssc.109	Q03333	445533
C	*PCSK4*	*Proprotein convertase subtilisin/kexin type 4*	complete	Ssc.47037	Not Found	100626523
C	*PCSK5*	*Proprotein convertase subtilisin/kexin type 5*	incomplete	Ssc.43614	Not Found	100519237
C	*PCSK6*	*Proprotein convertase subtilisin/kexin type 6*	incomplete	Ssc.73551	F1RZ92	100152144
C	*PCSK7*	*Proprotein convertase subtilisin/kexin type 7*	complete	Ssc.5628	F1SJT0	100523009
C	*PCSK9*	*Proprotein convertase subtilisin/kexin type 9*	complete	Ssc.84357	Not Found	100620501

^a^ P: prohormone gene, C: prohormone convertase gene.

^b^ Genome sequence found: complete or incomplete in the pig genome assembly, found in the Traces archive, or Not Found in any genome repository.

^c,d,e^ Identifiers in the UniGene, UniProt and Gene databases.

Table 2 summarizes the evidence from various repositories supporting the 95 unique pig prohormone genes and 2 alternative sequences detected in this study. The genome-predicted prohormone genes are supported by 66 UniProt entries (Table 1) including 47 sequences that have evidence at the protein level of which 39 have complete protein sequence and 8 have partial protein sequence. Additionally, 81, 91 and 19 prohormone genes detected are supported by transcript evidence from UniGene, Gene, and UniProt, respectively. Two genes, *apelin* (*APEL*) and *PROK2*, are supported by evidence in UniProt and by EST records unassigned to a UniGene cluster. UniProt supporting information includes 2 entries from alternatively spliced calcitonin genes, *preprocalcitonin gene-related peptide* and *calcitonin-2*, that have evidence at the protein and transcript levels, respectively. However, the *calcitonin 2* is not assigned to a UniGene cluster or NCBI Gene identifier because no EST matches the unique region of the reported sequence. The UniProt entry for *prothyroliberin* (*TRH*) refers only to the 3-amino acid thyroliberin peptide however, no pig EST has been reported. Only 8 genome predictions do not have supporting evidence in UniProt with 5 of these have supporting transcript evidence. Complete protein sequences were predicted for *insulin-like 5* (*INSL5*) and *endothelin-2* (*EDN2*) and an incomplete protein sequence was predicted for *prolactin-releasing peptide* (*PRRP*) genes. The nucleic and protein sequence of *EDN2* have been reported [22] but this information is not present in public databases. Of the 23 UniProt predicted sequences, 14 (7) entries have (do not have) transcript support. There are 2 UniProt predicted sequences that correspond to genes that are absent in the pig genome studied. A partial match to *UCN1* was found in the trace archives although this gene was not found in the pig genome assembly studied and lacks of external validation. *Progonadoliberin-2* (*GON2*) was not found in the current pig genome assembly although this gene was detected in earlier assembly versions and trace archives. This gene has no current experimental evidence outside homology to other mammalian species. The apparent lack of *UCN1* and *GON2* in the assembly and fragment evidence of other prohormones is most likely due to poor coverage of the genomic regions where these prohormone genes are located.

**Table 2:** Distribution of the prohormone gene predictions across UniProt and UniGene resources

		**UniProt evidence**^**1**^				
**Genome**^**2**^	**UniGene**^**3**^	**Protein**	**Partial**	**Transcript**	**Predicted**	**Not Found**
complete	Present	38	7	17	14	3
complete	Not Found	0	1	1	7	2
fragment	Present	1	0	1	0	2
fragment	Not Found	0	0	0	0	1
Not Found	Not Found	0	0	0	2	0

^1^ UniProt Evidence: “type of evidence that supports the existence of the protein”; Protein : complete protein sequence; Partial: incomplete protein sequence such as presence of a peptide; Transcript: “existence of a protein has not been strictly proven but there is expression data (such as existence of cDNAs, RT-PCR or Northern blots) that indicate the existence of a transcript.”; Predicted: Complete or partial sequence of the protein has been predicted; Not Found: no match found in the UniProt database.

^2^ Genome: prediction of the protein sequence from the genome assembly: complete denotes full sequence, fragment denotes incomplete prediction and Not Found denotes no match.

^3^ UniGene Present or Not Found denote whether the gene had any EST evidence or not, respectively.

At least four calcitonin genes, also known as calcitonin receptor-stimulating peptide genes, were identified with 2 genes exhibiting alternative splicing. The genome assembly permitted the assignment of the UniProt *pre-procalcitonin* (A6P7L6) and *preprocalcitonin gene-related peptide* (A6P7L7) entries to the same gene that also produces the UniProt calcitonin (*CALC*, [Swiss-Prot:P01259]) and calcitonin gene-related peptide (*CALCA*, [Swiss-Prot:P30880]) peptides, respectively. This alternatively spliced gene corresponds to *CALCA* gene found in other mammalian species.

The bioinformatics gene prediction pipeline confirmed that the separate Q766Y6 and A0A761 UniProt entries are alternatively spliced variants of the same *calcitonin receptor-stimulating peptide 3* (*CRSP3*) gene as initially reported by Rezaeian et al. [23]. A single genome match was identified for *calcitonin receptor-stimulating peptide 2* (*CRSP2*, Q766Y7). While the *calcitonin receptor-stimulating peptide 1* (*CRSP1*) gene has been reported, the actual gene may be inaccurately assembled in the 10.2 genome release because the region appears to contain a small duplication leading to 2 starting locations. Further supporting this argument, a conserved 19 amino acid region in all calcitonin receptor-stimulating peptide-related protein sequences matched to an additional 5th genome site which was part of a discontinued NCBI Gene entry (Gene ID 100624618). There is insufficient information to conclude whether there is a separate coding gene involved or an assembly-related problem.

*Neuropeptide S* (*NPS*) is a potential 12th prohormone gene discovered by the bioinformatics gene prediction pipeline. Two genome matches on different chromosomes for the *NPS* gene were identified. However, the complete identity of the matched between the predicted sequence and chromosomal locations implied that this was an assembly error rather than a duplication event. Although UniProt has an partial pig *NPS* entry (B5M997), the genome predicted protein sequence was more similar to the bovine sequence, as expected, than the present partial UniProt pig sequence. The pig *NPS* protein and nucleic sequences were found to be 100% and 99% identical, respectively, to the rabbit sequence ([GenBank:EU978456]). The similarity between the UniProt pig and rabbit sequences was also evident in the phylogenetic relationships among *NPS* sequences reported by Yao et al. [24]. These findings call into question the present pig *NPS* entry in UniProt. 

### Prohormone genes previously unreported in pig

The genome search identified 11 prohormone genes that do not have empirical confirmation in the UniProt, UniGene or Gene databases (Table 1). These genes are *ADM2*, *CORT*, *INSL5*, *OX26*, *PROK2*, *PRRP*, *NPS*, *TIP39*, *UCN1*, *UCN2*, *UCN3*, and *UTS2B*. Only inferred sequences are available for *ADM2* and *CORT* in UniProt and the current pig *NPS* entry is identical to the rabbit *NPS*. There is evidence for mammalian homologs of all these genes in UniProt. The protocol followed to identify these genes included a high percentage of identities and similarities with a minimum percentage of mismatches and gaps and conservation of the region encompassing the potential neuropeptide. *Intermedin* or *adrenomedullin 2* (*ADM2*) is part of the calcitonin family of peptides and has effects similar to those of *adrenomedullin* (*ADML*). In humans, *intermedin* causes hypotension when given peripherally and augments blood pressure and causes sympathetic activation when given to the central nervous system [25]. This neuropeptide induces prolactin release, has anti-diuretic and natriuretic properties and reduces food intake. The amino acid sequences of *CORT* and *somatostatin* (*SMS*) are highly similar and both reduce neuronal activity. In addition, *CORT* has unique roles such as induction of slow-wave sleep, reduction of locomotor activity, and activation of cation selective currents not responsive to *SMS*[26]. Although the function of *INSL5* is still being determined, high expression in the colon, as well as in the brain and hypothalamus, indicates roles in gut contractility and neuroendocrine signaling [27]. Likewise, the function of *OX26* is still being elucidated, although studies in chicken confirm the orexigenic, appetite stimulating activity of this neuropeptide [28]. Takayanagi and Onaka [29] demonstrated that *PRRP* plays a role in control of energy metabolism and stress response. Prokineticins are involved in tumorigenesis process (prostate, testicles, neuroblastoma, colon, and pancreas) acting as a growth factor for cancer cells, an angiogenic and a chemotactic factor for pro-inflammatory neutrophils [30]. *NPS* has anxiolytic-like effects (stress reduction) and can induce arousal and wakefulness [31]. *TIP39* and the corresponding receptor form a neuromodulator system and the anatomical distribution indicates a role in limbic, endocrine, viscerosensory, and auditory functions. This system has been postulated as potential drug target in anxiety, depression and chronic pain management [32]. Urocortins and their receptors has been found in the central nervous, digestive, reproductive, cardiovascular, immune and endocrine systems, suggesting a variety of roles including cardiovascular activity and cell survival [33]. *UTSB2* is a paralog of *urotensin 2* (*UTS2*) that exerts similar biological effects including relaxation of muscles and reduction of blood pressure [34].

### Pig prohormone convertase genes

The sequence of 8 prohormone convertase genes and the *7B2* facilitator gene also known as *secretogranin 5* (*SCG5*) were identified in the pig genome (Table 1). The UniProt and Gene databases only had supporting evidence for *PCSK1*, *PCSK2*, and *7B2*. Six additional prohormone convertase genes (*furin*, *PCSK4*, *PCSK5*, *PCSK6*, *PCSK7*, and *PCSK9*) that were previously unreported or not based on empirical evidence were identified. Only transcript evidence supports the 8 prohormone convertase genes, meanwhile protein evidence is available for *7B2*. Dai et al. [35] isolated *PCSK1* from the ovary cDNA library of a pregnant sow and Renegar et al. [36] detected *PCSK1* in the corpus luteum and brain of pregnant sows. Also, mRNA from *PCSK1* and *PCSK2* has been identified in the pituitary neurointermediate lobes of pigs [37]. Among the prohormone convertases, *furin*, *PCSK4*, *PCSK5*, *PCKS6* and *PCSK7* do not have UniGene entries. The present catalogue enhances the currently limited work on pig prohormone convertases.

### Functional characterization of the pig prohormone and prohormone convertase genes

Analysis of the large number of microarray gene expression experiments enabled the first comprehensive characterization of the role of prohormone and prohormone convertase genes in biological processes in the pig. The results from these analyses augmented the understanding of the role of these genes on reproduction, health, growth, and other traits of importance to biomedical research and agricultural production.

The query of Affymetrix Porcine Genome Array identified 77 probes representing 56 prohormone and 3 prohormone convertase genes. Table 3 lists the total number of differentially expressed probes (*P*-value < 0.005) within the seven experimental classes considered. A detailed distribution of the differential expression level of each probe and experiment is provided [see Additional file 3. A discussion of the findings for the 3 groups with highest number of differentially expressed probes (immune-related, embryo and placenta, and brain and central nervous system) is presented below. Although neuropeptides expressed in the brain and the immune system interact with circulating cytokines to support two-way communications between the brain and immune system [38], we describe the profiles of prohormones in immune-related tissues separately from the brain and central nervous system tissues to facilitate the interpretation of results.

**Table 3:** **Differentially expressed prohormone and prohormone convertase genes (*P*-value < 0.005) across 35 microarray experiments by tissue class**

**Symbol**	**Probe**^**a**^	**Imm.**^**b**^	**Emb.**	**CNS**	**Repro.**	**Musc.**	**Fat**	**Gut**	**Total**
**Prohormone**									
*ADM5*	Ssc.26627.1.A1_at	0	0	1	0	0	0	0	1
*ADML*	Ssc.314.1.S1_at	2	0	1	0	1	0	1	5
*ANF*	Ssc.16245.1.S1_at	0	0	0	0	1	0	0	1
*ANFB*	Ssc.629.1.S1_at	0	0	0	0	1	0	0	1
*ANFC*	Ssc.23867.1.A1_at	0	1	1	0	0	0	0	2
*AUGN*	Ssc.22487.1.S1_at	2	0	0	0	1	1	0	4
*CART*	Ssc.15900.1.S1_at	0	1	1	0	0	0	0	2
*CCKN*	Ssc.717.1.S1_at	1	0	0	1	1	0	0	3
*CMGA*	Ssc.4653.1.S1_at	0	0	0	0	0	1	1	2
*COLI*	Ssc.14556.1.S1_at	0	1	0	0	0	0	0	1
*CRSP1*	Ssc.3741.1.S1_at	0	0	0	0	0	0	0	0
*CRSP2*	Ssc.18558.1.S1_at	0	1	0	0	0	0	0	1
*CRSP3*	Ssc.17879.1.S1_at	1	0	0	0	0	0	0	1
*EDN1*	Ssc.9364.1.S1_at	2	0	0	0	0	1	0	3
*GALA*	Ssc.713.1.S1_at	1	1	0	0	0	0	1	3
*GALP*	Ssc.4875.1.S1_at	1	1	0	0	1	0	0	3
*GAST*	Ssc.644.1.S1_at	0	1	0	0	0	0	0	1
*GHRL*	Ssc.440.1.S1_at	0	0	0	0	0	0	0	0
*GLUC*	Ssc.17225.1.S1_at	0	1	0	1	0	0	1	3
*GON1*	Ssc.16310.1.S1_at	1	1	0	0	0	0	0	2
*HEPC*	Ssc.376.1.S1_at	0	0	0	0	0	0	0	0
*IAPP*	Ssc.8324.1.A1_at	0	1	0	0	0	0	0	1
*IGF1*	Ssc.16231.1.S1_a_at	1	0	1	0	0	0	0	2
	Ssc.16231.2.A1_a_at	0	0	0	0	0	0	0	0
	Ssc.16231.3.S1_a_at	0	0	1	0	0	0	0	1
*IGF2*	Ssc.9365.1.S1_at	1	0	0	0	0	0	0	1
	Ssc.9365.2.S1_a_at	1	1	0	0	0	1	0	3
	Ssc.9365.3.S1_a_at	1	0	0	0	0	0	0	1
	Ssc.9365.3.S1_x_at	0	0	0	0	0	0	0	0
	Ssc.9365.4.S1_a_at	0	1	0	0	0	0	0	1
	Ssc.9365.5.A1_at	1	0	0	0	0	0	0	1
	Ssc.9365.5.S1_at	1	1	0	0	0	0	0	2
	Ssc.9365.5.S1_a_at	0	0	0	0	0	1	0	1
	Ssc.9365.6.A1_a_at	0	0	0	0	0	0	0	0
	Ssc.9365.6.A1_x_at	0	0	0	0	0	0	0	0
	Ssc.9365.6.S1_x_at	1	0	1	0	0	0	0	2
	Ssc.9365.7.A1_x_at	0	0	0	0	0	0	0	0
*INS*	Ssc.583.1.S1_at	0	0	0	0	0	0	0	0
*INSL3*	Ssc.11990.1.S1_at	0	1	0	0	0	0	0	1
*MCH*	Ssc.3287.1.S1_at	0	0	0	0	0	0	0	0
*MOTI*	Ssc.714.1.S1_at	0	0	0	0	0	0	0	0
*NEU1*	Ssc.15668.1.A1_at	0	0	0	0	0	0	0	0
*NEU2*	Ssc.4210.1.S1_at	0	0	0	0	1	0	0	1
*NMB*	Ssc.2083.1.A1_at	1	0	0	0	0	0	0	1
*NMU*	Ssc.12508.1.A1_at	1	0	0	0	0	0	0	1
*NPW*	Ssc.15796.1.S1_at	0	1	0	0	0	0	0	1
*NPY*	Ssc.15981.1.A1_at	1	1	0	0	1	0	0	3
	Ssc.15981.1.S1_at	0	2	1	0	0	0	0	3
*OREX*	Ssc.15983.1.S1_at	0	0	0	0	0	0	0	0
*PACA*	Ssc.27598.1.S1_at	0	1	0	0	0	0	0	1
*PAHO*	Ssc.456.1.S1_at	0	1	0	0	0	0	0	1
*PCSK1N*	Ssc.17429.1.S1_at	0	1	0	1	0	0	0	2
*PDGFA*	Ssc.6173.3.S1_a_at	1	0	1	0	0	0	1	3
*PDYN*	Ssc.121.1.S1_at	0	1	1	0	0	0	0	2
*PENK*	Ssc.11281.1.A1_at	0	1	0	1	0	1	1	4
	Ssc.11281.2.S1_at	1	1	0	0	0	0	0	2
*PNOC*	Ssc.15910.1.A1_at	0	0	0	0	0	0	0	0
	Ssc.15910.1.S1_at	0	0	0	0	0	0	0	0
*PTHR*	Ssc.9991.1.S1_at	0	1	1	2	0	0	0	4
*PTHY*	Ssc.668.1.S1_at	0	1	0	0	0	0	0	1
*REL1*	Ssc.162.1.S1_at	1	1	0	0	0	0	0	2
*SCG1*	Ssc.15718.1.A1_at	1	1	0	0	0	0	1	3
*SCG2*	Ssc.13645.1.A1_at	1	0	0	0	0	1	1	3
*SCG3*	Ssc.6770.1.A1_at	1	1	0	1	0	0	0	3
*SECR*	Ssc.710.1.S1_at	0	1	0	0	0	0	0	1
*SMS*	Ssc.19520.1.A1_at	1	1	0	0	1	0	0	3
*TKN1*	Ssc.18075.1.A1_at	0	0	0	0	0	0	0	0
	Ssc.18075.2.S1_at	0	1	0	0	0	0	0	1
*TKN4*	Ssc.23153.1.S1_at	0	0	0	0	0	0	0	0
*TKNK*	Ssc.19565.1.S1_at	0	0	0	0	0	0	0	0
	Ssc.19565.2.A1_at	0	0	0	0	0	0	0	0
*UTS2*	Ssc.437.1.S1_a_at	0	1	0	0	0	0	0	1
*VEGFC*	Ssc.12790.1.A1_at	1	1	1	0	1	0	1	5
*VEGFD*	Ssc.29289.1.A1_at	1	1	0	0	0	0	0	2
Total		30	35	12	7	10	7	9	110
**Prohormone Convertase**									
*PCSK1*	Ssc.141.1.S1_at	1	1	0	0	0	0	1	3
*PCSK2*	Ssc.109.1.S1_at	0	0	0	0	0	0	0	0
*PCSK7*	Ssc.5628.1.S1_at	1	1	0	0	0	0	1	3
Total		2	2	0	0	0	0	2	6

^a^Affymetrix microarray gene probe identifier.

^b^ Experiment classes: Imm: primary immune-response tissues, Emb: embryo and placenta, CNS: brain and central nervous system, Repro: reproduction, Musc: muscle, fat, and gut.

#### Immune-related profiling

Several studies have demonstrated that prohormone genes play an important role in pig immune response [39]. This was evidenced by the high number of differentially expressed prohormone and prohormone convertase genes (24 genes) among experiments that evaluated immune-response in blood, spleen, and lymph nodes (Table 3). Differentially expressed genes were: *ADML*, *augurin* (*AUGN*), *cholecystokinin* (*CCKN*), *CRSP3*, *endothelin-1* (*EDN1*), *galanin* (*GALA*), *galanin-like peptide* (*GALP*), *progonadoliberin-1* (*GON1*), *insulin-like growth factor I* (*IGF1*), *insulin-like growth factor II* (*IGF2*), *neuromedin-B* (*NMB*), *neuromedin-U* (*NMU*), *neuropeptide Y* (*NPY*), *platelet-derived growth factor subunit A* (*PDGFA*), *proenkephalin-A* (*PENK*), *prorelaxin 1* (*REL1*), *secretogranin-1* (*SCG1*), *secretogranin-2* (*SCG2*), *secretogranin-3* (*SCG3*), *SMS*, *vascular endothelial growth factor C* (*VEGFC*), *vascular endothelial growth factor D* (*VEGFD*), *PCSK1* and *PCSK7*.

In general, prohormone genes were under-expressed in pigs under immune challenge relative to the un-challenged controls. *AUGN* was differentially expressed in two experiments; GSE7313 [40] that profiled lymph nodes and GSE14790 [41] that profiled blood. In GSE14790, 7 day-old pigs were inoculated with porcine circovirus type 2 (PCV2), a virus that is widely spread across pig farms, and gene expression was profiled at 0, 7, 14, 21 and 29 dpi. *AUGN* was over-expressed in un-inoculated pigs at 29 dpi relative to 7 dpi, regardless of inoculation and relative 21 dpi inoculated pigs (*P*-value < 2.5 × 10^-4^). Both contrasts indicate that the expression of *AUGN* increases with age and this trend is slower in pigs infected with PCV2. In GSE7313, the gene expression of seven week old piglets inoculated with *Salmonella Typhimurium* was profiled at 8 hours post inoculation (hpi), 24 hpi, 48 hpi, and 21 days post inoculation (dpi). *AUGN* was over-expressed at 21 dpi relative to 24 hpi and 48 hpi (*P*-values < 6.8 × 10^-5^ and 2.7 × 10^-6^, respectively). Consistent with the differential expression in relation to immune-response observed in this study, *AUGN* is a putative tumor suppressor gene and is down-regulated in many cancers [42].

*IGF2*, a member of the insulin family and is involved in development and growth, was differentially expressed across immune-related experiments. *IGF2* was represented by 12 probes in the microarray platform and 6 probes were differentially expressed across experiments. Five probes (Ssc.9365.1.S1_at, Ssc.9365.2.S1_a_at, Ssc.9365.5.A1_at, Ssc.9365.5.S1_at, Ssc.9365.6.S1_x_at) and one probe (Ssc.9365.3.S1_a_at) were differentially expressed in experiments GSE14790 [41] and GSE7314 [43], respectively. In GSE14790, *IGF2* was under-expressed in non-inoculated piglets at 7 dpi relative to inoculated pigs at various days (*P*-value < 1.7 × 10^-5^, fold change = 0.71). In experiment GSE7314, *IGF2* was over-expressed in pigs inoculated with *Salmonella choleraesuis* at 21 dpi relative to non-inoculated pigs (*P*-value < 8.1 × 10^-4^). These results are consistent with reports that *IGF2* is down-regulated in pigs immune-challenged with lipopolysaccharide [44].

*SCG1*, *SCG2*, *SCG3*, members of the secretogranin family, exhibited differential expression among immune-challenge experiments consistent with the known association of these genes with cell activation, cytotoxicity and microbial defense [45]. Probes on all 3 SCGs exhibited differential expression on two immune-related experiments. *SCG1* and *SCG2* are differentially expressed in GSE14790 while *SCG3* was differentially expressed in GSE11787 [21]. In GSE11787 *SCG3* was under-expressed in inoculated pigs relative to controls (*P*-value < 1.2 × 10^-3^, fold change = 0.33). These results are consistent with the lack of synthesis of endogenous granins in rat PC12 cells infected with recombinant vaccinia viruses [46]. In GSE14790, *SCG1* and *SCG2* were under-expressed in pigs inoculated with PCV2 relative to un-inoculated control pigs (*P*-value < 1.8 × 10^-4^, fold change = 0.88 and *P*-value < 1.5 × 10^-5^, fold change = 0.93, respectively).

Two members of the vascular endothelial growth factor family, *VEGFC* and *VEGFD*, were under-expressed in PCV2 inoculated pigs relative to control pigs (*P*-value < 1.8 × 10^-5^, fold change = 0.62) and also under-expressed at early stages (7 dpi) relative to later stages (19 and 29 dpi) in GSE14790. In agreement with these findings, a loss of endothelial growth factor transcription and increase in pro-inflammatory indicators were reported in the endometrial lymphocytes of pigs at sites of fetal arrest [47].

*NPY* (probe Ssc.15981.1.A1_at) was under-expressed in PCV2-inoculated pigs relative to control pigs (*P*-value < 6.6 × 10^-4^) and, within infection level, *NPY* was under-expressed at earlier stages relative to 29 dpi in GSE14790. Consistent with these findings, the levels of *NPY* mRNA decreased in the blood of rats treated with vinblastine, an anti-cancer drug known to decrease the number of white blood cells of the immune system involved in defense [48]. Similarly, *NPY* was found to decrease in cattle infected with Bovine Spongiform Encephalopathy [49].

*ADML* was differentially expressed in GSE14758-D and GSE7314. In GSE14758-D [41], *ADML* was under-expressed in the mediastinal lymph nodes of PCV2-infected pigs relative to control pigs at 29 dpi (*P*-value < 1.7 × 10^-3^, fold change = 0.6). Whereas, in GSE7314 *ADML* was over-expressed at 48 dpi in the blood of pigs inoculated with *Salmonella choleraesuis* relative to controls (*P*-value 4.8 × 10^-3^). The latter result is consistent with the up-regulation of *ADML* gene expression and increases in systemic circulatory concentrations of *ADML* in response to the onset and progression of trauma, infection, and sepsis [50]. The apparent inconsistency between both experiments may be associated with the differential effects that *ADML* has on cellular metabolism, immune function, endocrine function, and cardiovascular function.

Of the 3 prohormone convertases available in the microarray platform, *PCSK1* and *PCSK7* were significantly differentially expressed (*P*-value < 1.3 × 10^-3^) and *PCSK2* was marginally significantly differentially expressed (*P*-value < 6.5 × 10^-3^) in GSE14790. *PCSK1* was under-expressed in PCV2-inoculated pigs already at 7 dpi relative to 29 dpi, regardless of inoculation at the later stage (*P*-value < 5.7 × 10^-5^). Likewise, *PCSK7* is under-expressed in PCV2-inoculated pigs relative to controls already at 7 dpi (*P*-value < 4.2 × 10^-4^) and, within controls, *PCSK7* was under-expressed at early stages (7 dpi, 21 dpi) relative to 29 dpi (*P*-value < 4.1 × 10^-4^). These results are in agreement with similar findings that *furin*, another prohormone convertase, was dysregulated in the immune cells of advanced human atherosclerotic plaques [51] and imply that prohormone convertase, like prohormone genes, are down regulated under immune challenges.

#### Embryo and placenta profiling

In GSE18641 [52], *IGF2* (probe Ssc.9365.2.S1_a_at) was over-expressed in pregnant sows relative to non-pregnant sows (*P*-value < 2.7 × 10–3, fold change 1.23). In GSE12705 [20], *IGF2* (probes Ssc.9365.4.S1_a_at and Ssc.9365.5.S1_at) was over-expressed in earlier stages (day 11 spherical and day 11 and 12 tubular) relative to later stage (day 12 and 14 filamentous) conceptuses (*P*-value < 2.1 × 10^-4^). This *IGF2* profile is supported by Pantaleon et al. [53] that showed that *IGF2* is needed in order for mouse embryos to progress from early stages to blastocyst stages. Gupta et al. [54,55] reported that the expression of the embryo survival related gene *IGF2* increased with the addition of nonessential amino acids or phytohemaglutinin in pig embryos and blastocysts, respectively.

Both *PENK* probes were over-expressed in tubular and spherical conceptuses relative to filamentous conceptuses (*P*-value < 2.6 × 10^-6^) in experiment GSE12705 [20]. This is consistent with results that found *PENK* mRNA to increase linearly during gestation in the hippocampus of pigs [56]. *PTHR* was under-expressed in tubular and spherical relative to filamentous conceptuses (*P*-value < 6.4 × 10^-7^, fold change = 0.02) in experiment GSE12705. This finding is supported by reports that *PTHR* is present in higher concentrations in fetal pigs than in sows [56]. *VEGFC* is a representative of the vascular endothelial growth factor family of prohormones that have an important role in the survival and mitogenesis of endothelial cells and lymphaniogenesis and angiogenesis of embryos [57]. *VEGFC* was over-expressed in pregnant sows relative to non-pregnant sows (*P*-value < 7.8 × 10^-4^) in experiment GSE18641 [52]. This finding is supported by a study in the chicken, demonstrating that the chorioallantoic membrane (analogous to the placenta in mammals) contained growth of embryonic microvessels stimulated by *VEGFC*[58]. The expression profile is also supported by the finding that in mice embryos, *VEGFC* is required for successful lymphatic vasculature development and lymphatic endothelial cell migration [59].

#### Brain and central nervous system

Eleven differentially expressed prohormone genes were identified in experiments concerning the hypothalamus, thyroid, and olfactory bulb (neuroblasts). These genes are *Adrenomedullin-5* (*ADM5*), *ADML*, *C-type natriuretic peptide* (*ANFC*), *cocaine and amphetamine regulated transcript protein* (*CART*), *IGF1*, *IGF2*, *NPY*, *platelet-derived growth factor subunit A* (*PDGFA*), *prodynorphin* (*PDYN*), *PTHR*, and *VEGFC*.

*ADML* was over-expressed in the immortalized porcine olfactory bulb neuroblasts relative to the non-neural epithelial cells (*P*-value < 2.2 × 10^-6^, fold change > 10) in experiment GSE16855 [60]. This result is supported by a previous study that found that *ADML* is important for regulation of proliferation and differentiation of neural stem/progenitor cells using the mouse olfactory bulb [61].

*IGF1* was over-expressed in the neuroblasts relative to non-neural epithelial cells (average *P*-value < 5 × 10^-7^, fold change > 10) in experiment GSE16855. This result is supported by a study in chickens showing that *IGF1* was expressed in the olfactory bulb [62]. Also, *IGF2* (probe Ssc.9365.6.S1_x_at) was consistently over-expressed in the hypothalamus of male Iberian pigs relative to all other seven breed-gender combinations (on average, *P*-value < 2.3 × 10^-4^, fold change = 2.42) in experiment GSE14739-H [63,64]. *NPY* was over-expressed (*P*-value < 8.1 × 10–4, fold change = 7.94) in neuroblasts relative to non-neuronal cells in GSE16855. This result is consistent with reports that the olfactory bulb exhibit high levels of immunoreactive *NPY* in the brain of pigs [65] and that *NPY* may inhibit excitatory neurotransmission in the rat olfactory bulb [66]. *VEGFC* was over-expressed in neuroblasts relative to non-neuronal cells (*P*-value < 1.5 × 10^-9^, fold change > 10) in experiment GSE16855. This result agrees with a 30% increase in dividing neuroblasts in olfactory bulb in culture stimulated with *VEGFC* compared to controls reported by Le Bras et al. [67]. *PTHLH* was under-expressed in neuroblasts relative to non-neuronal cells (*P*-value < 2.6 × 10^-4^, fold change = 0.20) in GSE16855. This finding is consistent with reports that *PTHLH* may be a negative regulator in the differentiation of chondrocytes [68]. *PDGFA* was over-expressed in neuroblasts relative to non-neuronal cells (*P*-value < 1.2 × 10^-4^) in experiment GSE16855. Related to this result, Fressinaud et al. [69] reported that platelet-derived growth factors increase the glutamine synthetase activity in astrocytes in the brain.

### Prediction of cleavage sites in pig prohormones

All 97 prohormone sequences were used to predict cleavage and confirm the prediction against known or predicted cleavage sites. These sequences were inferred to have 228 cleavage sites that resulting in a 14.6% prevalence rate (proportion of possible sites that are cleaved). Most sites were cleaved at an arginine (R) such that the most frequently cleaved motifs were xxKR (71%), RxxR (34%) and xxRR (41%), where x denotes any amino acid and K denotes lysine. There were 5% (38) C-terminal single R sites that were cleaved without a basic amino acid in the second and fourth positions preceding the cleavage site (P2 or P4 locations, respectively).

The performance of the cleavage prediction models is presented in Table 4. The correct classification rate ranged from 82% to 92% indicating that a large proportion of the sites were accurately predicted across all models. The human cleavage prediction models had the best performance for most of the statistics followed by the mammalian model. The Known Motif model provided the highest number of true positive predictions but also the highest number of false positive predictions. The Known Motif model provided the highest sensitivity, 77%, indicating more than three quarters of the cleaved sites were correctly predicted as cleaved. However this model also provided the highest number of false positive predictions. Consequently the Known Motif positive predictive power was 35% indicating that, on average, only 35% of sites predicted to be cleaved are expected to be true cleavage sites.

**Table 4:** Performance of various cleavage prediction models to predict cleavage in pig prohormones

**Performance**	**Known**	**Mammalian**	**Human**	**Logistic**	**Human**	**ANN**^**d**^
**Criteria**^**a**^	**Motif**	**Logistic**	**AA**^**b**^	**AA Prop.**^**c**^	**AA**	**AA Prop.**
True Positives	181	165	160	158	164	167
True Negatives	1520	1640	1724	1670	1735	1747
False Positives	329	209	125	179	114	102
False Negatives	54	70	75	77	71	68
Correct Classification	0.8162	0.8661	0.904	0.8772	0.9112	0.9184
Sensitivity	0.7702	0.7021	0.6809	0.6723	0.6979	0.7106
Specificity	0.8221	0.887	0.9324	0.9032	0.9383	0.9448
Positive predictive power	0.3549	0.4412	0.5614	0.4688	0.5899	0.6208
Negative predictive power	0.9657	0.9591	0.9583	0.9559	0.9607	0.9625
Correlation	0.4358	0.4856	0.5645	0.4944	0.5919	0.6184
AUC	0.8006	0.847	0.86	0.8186	0.8589	0.8802

^a^ Performance criteria. True positives: number of correctly predicted cleaved sites; True negatives: number of correctly predicted non-cleaved sites; False positives: number of incorrectly predicted cleaved sites; False negatives: number of incorrectly predicted non-cleaved sites; Correct classification rate: number of correctly predicted sites divided by the total number of sites; Sensitivity (one minus false positive rate): number of true positives divided by the total number of sites cleaved; Specificity (one minus false negative rate): number of true negatives divided by the total number of sites not cleaved; Positive predictive power: number of true positives divided by the total number of sites predicted to be cleaved; Negative predictive power: number of true negatives divided by the total number of sites predicted to not be cleaved; Correlation coefficient: Mathew’s correlation coefficient between observed and predicted cleavage; and AUC: Area under the receiver operator characteristic or ROC curve relating sensitivity and 1-specificity.

^b^ AA: models trained only on amino acids.

^c^ AA prop: models trained with amino acids combined with the physicochemical properties of amino acids.

^d^ ANN: artificial neural network approach.

The human models provided the highest number of true negatives resulting in the best model performance compared to the Known Motif and mammalian models. The human artificial neural network models had approximately 60% positive predictive power indicating that most sites predicted as cleaved are expected to be true positives. Although the human logistic models had lower sensitivity than their artificial neural network counterparts, the differences with the human artificial neural network model were only 4 cleaved and 11 non-cleaved sites. The high performance of the human models suggests that the cleavage of prohormones that result in potential biologically active neuropeptides in the pig is similar to humans. Noteworthy is that the mammalian model was trained on 51 mammalian prohormones that included 8 pig prohormones. This model provided slightly more true positive predictions and a higher sensitivity than the human logistic model. However, the mammalian model had noticeably more false positive predictions than the human logistic model resulting in lower performance in the other accuracy measures.

The comparison of results across models also provides information on the accuracy of the cleavage assignment, prediction accuracy and potential for a gene to produce bioactive peptides. For 10 prohormones, at least 5 of the models did not predict any cleavage site. However, it must be noted that 4 of the prohormones (*ANF*, *GHRL*, *IGF1* and *PDGFD*) are likely to have sites cleaved by proteases other than prohormone convertases. For example, *ANF* is cleaved by *corin, serine peptidase* (*CORIN*) [70].

Genes with no predicted cleavage or assigned cleavage that differ from other species can be used to identify proteins are not cleaved to form smaller peptides. There is no evidence for cleavage of *UCN2* and *UCN3* to produce mature peptides in mammals [71]. All models failed to predict two cleavage sites in *TOR2X*. The first site, an N-terminal dibasic ‘RK’, is known to be rarely cleaved across species [72]. The second site is a cleavage found in humans that forms alpha- and beta-salusin but this site may not be cleaved in the pig since the pig sequence, like the bovine sequence, only has a single basic site instead of the human dibasic ‘RR’ site. Similarly for *TKN4*, the genomic prediction and supporting EST data indication a change from an R in other species to a glycine amino acid in the pig sequence that may prevent the formation of the ‘Hemokinin’ peptide.

The pig *CRSP2* protein sequence lacks the ‘KR’ and a C-terminal cleavage site that are cleaved in human *CALC* and *CALCB* genes to produce Calcitonin gene-related peptide 1 and Calcitonin gene-related peptide 2. Therefore it is unlikely that pig *CRSP2* would provide these calcitonin peptides. The assigned cleavages in the *RES18* protein are necessary to provide a potential *triskadecapeptide* peptide reported by Bloomquist et al. [73]. This potential peptide has flanking dibasic cleavage sites in the mouse and rat but this peptide has not been experimentally confirmed. The corresponding region in human, bovine and pig sequences are monobasic and lack common PC cleavage motifs suggesting that these species probably cannot form this peptide.

Examination of the potential cleavage sites in *IAPP* indicated that a mutation from R to Q in the N-terminal cleavage site is necessary to produce the Islet amyloid polypeptide. Examination of the corresponding ESTs indicated that 2 swine ESTs ([GenBank:AJ649149] and [GenBank:AJ649469]) were 100% identical to the rat genome and consequently invalid sequences. Two other ESTs ([GenBank:EW569366], [GenBank:BF712755]) matched the region that supported the genomic prediction. The predicted protein sequence including the potential cleavages sites of the expected *IAPP* was less than 80% identical to other mammals sequences compared to typically over 85% identity between the human and most other mammalian sequences. Potter et al. [74] questioned the capability of *IAPP* to form amyloids after examining the functionality of a synthesized pig sequence based on the [GenBank:BF712755] EST sequence. The predicted prohormone sequence and cleavage prediction results also strongly suggest that the pig is unlikely to be able to form *IAPP*. This reflects the importance of proteomic studies involving cleavage to first determine that a species can produce a peptide.

## Conclusions

The pig is an important biomedical and agricultural research species. Results from the first genome-wide study of pig prohormone and prohormone convertase genes, functional annotation and prediction of prohormone cleavage have been presented. This study was enabled by the availability of the pig genome sequence and of 35 gene expression experiments that evaluated a wide range of conditions in pigs. These results offer more insights into the role of neuropeptides on biological processes such as reproduction, development, growth, and health and support targeted empirical confirmation. The bioinformatics pipeline used in this study can be used to identify prohormones or other sets of genes in species with similar sequence resources. Confirmatory insight into the pig prohormones can be expected from proteomic mass spectrometry studies.

Combining complementary bioinformatic resources, 95 prohormone genes, 8 prohormone convertases and one cleavage facilitator were discovered in the pig genome and raw sequence repositories. We uncovered 11 prohormone genes that have not been previously reported and one potentially incorrectly reported. The high performance of the models used to predict cleavage in the pig prohormones suggests that the prohormone cleavage in pigs is similar to humans. The analysis of 35 gene expression experiments identified various neuropeptide genes differentially expressed in immune-related tissues, embryo and placenta and the central nervous system including *AUGN*, *IGF2*, the family of *SCG*s, *NPY*, *ADM* and *ADML*, *PENK*, *PTHR*, and *VEGFC*. Experiments are required to confirm that the pig does not produce the bioactive neuropeptides *UCN2*, *UCN3*, *TOR2X*, *TKN4*, *IAPP*, and *CRSP2* as suggested by the cleavage prediction models.

## Methods

### Search for pig prohormone and convertase genes

A registry of approximately 100 candidate mammalian prohormone and convertase genes was built from public sequence databases (including NCBI Gene –release date September 2011 [75], UniGene [76] – release date April 13 2011, and UniProt [77]–release 2011_11 November 16, 2011) and a literature review [11-13,19,78-80].

Candidate genes were searched for in the pig genome 10.2 assembly using the bioinformatics pipeline developed by Southey et al. [11,13]. The protein sequence of each candidate gene in the registry was searched on the pig genome assembly using the TBLASTN, BLASTP and BLASTN programs from NCBI BLAST (version 2.18) [81] with default parameters settings (E-value < 10 and BLOSUM62 scoring matrix) and filtering disabled. In addition, sequences not used in the pig genome assembly (including unassigned genomic regions, whole genome shotgun sequencing and trace archives) were searched when there was no suitable BLAST match to a candidate gene or when the alignment to the genome assembly suggested a missing genomic region. This strategy allowed the annotation of genomic regions that were partly or not included in the assembly.

The BLAST matches were examined based on the alignment score and E-value to identify the most likely matches and genomic location of the corresponding prohormone. The identified pig genomic region that encompassed the BLAST match was further extended approximately 500 base pairs to the 5′ and 3′ ends of the match. Matches were also screened for alignments to multiple homologous prohormone genes that could indicate gene duplication events in the pig genome. The gene parsing tool Wise2 [82] was used to predict the protein sequence within the genome regions detected with BLAST. The genomic region was further extended when only a partial protein sequence was predicted. In this study, Wise2 compared the target protein (preference was given to pig protein sequences, followed by human, cattle and other mammals) to the pig genomic DNA sequence identified by BLAST to infer the gene structure based on a model that includes introns and frameshift errors. Each predicted gene was compared to the UniProt and NCBI Gene databases to assess the accuracy of the prediction based on previously reported pig genes. To further confirm the Wise2 predictions, the protein sequence predicted from the gene model was also compared to the corresponding published mammalian sequences using the multiple sequence alignment tool Clustalw [83]. The multiple sequence alignment maximized the likelihood of identifying homologous genes. The predicted sequences were also searched against the pig entries in the NBCI EST database to confirm the presence of the predicted protein sequence. The pig entries in the NCBI EST database was also used to complete the protein sequence when the genome coverage was incomplete.

### Functional annotation of the pig prohormone and convertase genes

A review of the pig microarray gene expression experiments available in the NCBI GEO database [84] indicated that the Affymetrix Porcine Genome Array GPL3533 [85] was the most commonly used platform. The UniGene database was searched for sequences that represent prohormone and prohormone convertase genes. This information was used to identify the probes representing prohormone and prohormone convertase genes in the Affymetrix Porcine Genome platform.

Thirty-five experiments that used the Affymetrix Porcine Genome platform were identified in GEO. Selected experiments had a minimum of 6 microarrays and a maximum of 80 microarrays. The sources and main features of these experiments are provided [see Additional file 4]. The wide range of selected microarray experiments available supported a comprehensive characterization of the association of prohormone and associated neuropeptide and convertase genes with various biological processes.

The experiments were grouped into 7 classes: primary immune-response tissues, embryo and placenta, brain and central nervous system, reproduction, muscle, fat, and gut. For experiments encompassing multiple tissues (GSE14739, GSE18359, GSE13528), the samples corresponding to each tissue were grouped and analyzed separately to facilitate the interpretation of results. The number of GEO experiments in each within each class were: immune: 6 (GSE7313, GSE7314, GSE11787, GSE17492, GSE14758-mediastinal lymph nodes, and GSE14790); embryo and placenta: 5 (GSE18467, GSE18641, GSE18343, GSE11853, and GSE12705); brain and nervous system: 5 (GSE16855, GSE12604, GSE14739-hypothalamus, GSE14739-thyroid, and GSE14739-adenohypophsis); reproduction: 2 (GSE11590, and GSE14739-gonads); muscle: 7 (GSE18653, GSE19275, GSE8974, GSE14643, GSE15211, GSE21096, and GSE16348-skeletal muscle); fat: 8 (GSE17309, GSE14373, GSE14739-fat, GSE9333, GSE18359-fat, GSE18359-liver, GSE13528-fat and GSE13528-liver); gut: 2 (GSE14357 and GSE15256).

The gene expression data were pre-processed and normalized using the Affy R package [86]. Steps included the log-2 transformation and GC-robust multichip average normalization of the gene expression measurements. All probes in the platform were analyzed using ANOVA to identify those that exhibited differential expression across the conditions studied. The false discovery rate [87] approach was used to adjust the statistical significance of the differential expression and account for multiple testing across all probes. The normalization, one or two-way ANOVA and multiple test adjustment of the results were done using Beehive [88].

### Prediction of cleavage sites

The location of the cleavage in pig prohormone proteins that would result in potentially active neuropeptides was predicted using NeuroPred [72]. Complete prohormone sequences from UniProt were used to predict cleavage in preference to the predicted sequences. In limited cases, EST sequences were combined with the genomic data and published partial sequences to predict the complete prohormone sequence. For example, for *Chromogranin-A* (*CMGA*), three glutamic acids were missing in the genome-based predictions that were present in the corresponding UniProt fragment sequence ([Swiss-Prot:P04404]) and EST sequence [GenBank:EW261315] permitted the prediction of the complete pig *CMGA* protein sequence. The location of the potential cleavage sites in the pig prohormones were inferred by homology to human data.

Complementary cleavage prediction models trained on confirmed cleavages from mammalian sequences [12,78,79] were used to predict cleavages in the pig prohormone sequences. These models included the known motif model that searches for sites with specific combinations of basic amino acid associated to cleavages reported in other species [78], mammalian logistic regression [79], and human logistic regression and artificial neural network models based on amino acids only or amino acids combined with the physicochemical properties of amino acids [12].

Known or predicted cleavage sites on all 97 prohormone sequences were used to assess the performance of the models to predict cleavage. The “observed” cleavage sites known or inferred from homology to other species based on a literature search [11-13,19,78-80] were compared to the cleavage sites predicted by the models. The counts of the true positives (number of correctly predicted cleaved sites), true negatives (number of correctly predicted non-cleaved sites), false positives (the number of incorrectly predicted cleaved sites) and false negatives (number of incorrectly predicted non-cleaved sites) or functions of the counts were used to assess the model performance. These measurements were used to compute the correct classification rate (number of correctly predicted sites divided by the total number of all sites), sensitivity (number of true positives divided by the total number of cleaved sites), specificity (number of true negatives divided by the total number of non-cleaved sites), positive predictive power (number of true positives divided by the total number of sites predicted to be cleaved), negative predictive power (number of true negatives divided by the total number of sites predicted to not be cleaved), Mathew’s correlation coefficient between observed and predicted cleavage. The area under the receiver operator characteristic or ROC curve relating sensitivity and 1 - specificity [78] was also calculated where area values lower than 0.7 indicate poor model performance.

## Competing interests

The authors declare that they have no competing interests.

## Authors’ contributions

KIP performed the search for prohormone and prohormone convertase genes in the UniProt, Gene, UniGene and ENSEMBL databases, identified the gene probes on the microarray platform, analyzed 35 microarray experiments, contributed to the interpretation of results, and drafted the manuscript. BRS located the prohormone and prohormone convertase genes in the pig genome assembly, trace archives and EST databases, predicted the genes from the genome sequence, compared the prediction to known sequences, contributed to the interpretation of results, and manuscript. JVS obtained funding for the study, contributed to the interpretation of the results, and reviewed the manuscript. SRZ obtained funding for the study, participated in its conception, coordination, interpretation of results, and helped write the manuscript. All authors have read and approved the final version of this manuscript.

## Supplementary Material

Additional file 1 **Table S2.** Prohormone and convertase genes identified across multiple pig genome resources. Click here for file

Additional file 2 Prohormone sequences and cleavage in NeuroPred. Click here for file

Additional file 3 **Table S3.** Statistical significance *P*-value corresponding to the comparison between groups within experiment. Click here for file

Additional file 4 **Table S1.** Main features of the 35 microarray experiments analyzed. Click here for file
