# Supplementary material for: First survey and functional annotation of prohormone and convertase genes in the pig
Source: BMC Genomics. 2012 Nov 15;13:582. doi: 10.1186/1471-2164-13-582 (PMC3499383; doi:10.1186/1471-2164-13-582)
Supplement: Additional file 1 — Table S2. Prohormone and convertase genes identified across multiple pig genome resources. [file 1471-2164-13-582-S1.doc]

**Supplementary Table 2.** Main features of the 35 microarray experiments analyzed to functionally annotate the prohormone and prohormone convertase genes.

| Experiment^a^ | Tissue | Class | Gender^b^ | Age | Comparison(s) | Reference |
| --- | --- | --- | --- | --- | --- | --- |
| GSE7313 | Mesenteric Lymph Nodes | Immune | NA | 7 weeks | Non-Infected, 8 hpi, 24 hpi, 48 hpi, 21 dpi | {{226 Wang,Y. 2007;}} |
| GSE7314 | Mesenteric Lymph Nodes | Immune | NA | 7 weeks | Non-Infected, 8 hpi, 24 hpi, 48 hpi, 21 dpi | {{289 Wang,Y. 2008;}} |
| GSE8974 | Endothelial Cells | Muscle | F | 1 week | Cultured, Regenerated | {{290 Lee,M.Y. 2010;}} |
| GSE9333 | Adipose Tissue | Fat | NA | 6-7 months | Korean Native Pig, Yorkshire | {{291 Moon,J.K. 2009;}} |
| GSE11590 | Oocytes | Reproduction | F | 42-44 hours | Sow, Gilt | {{227 Paczkowski,M. 2011;}} |
| GSE11787 | Spleen | Immune | M/F | 30 days | Haemophilusparasuis infected, non-infected | {{225 Chen,H. 2009;}} |
| GSE11853 | Placenta | Embryo and Placenta | F | 75 or 90 days after gestation | Erhualian, Large White, day 75, day 90 | {{228 Zhou,Q.Y. 2009;}} |
| GSE12604 | Corneal/Conjuctival clone | Brain and Nervous System | NA | NA | Corneal, Conjuctival clone | {{229 Majo,F. 2008;}} |
| GSE12705 | Embryo | Embryo and Placenta | F | 11, 12 or 14 days | Spherical, tubular, filamentous | {{224 Ross,J.W. 2009;}} |
| GSE13457 | Jejunum | Gut | NA | 21-35 days | day 21, 24, 28,35, animal, vegetable protein diet | {{311 Sun, Y.;}} |
| GSE13528-F | Fat | Fat | F | 6 months | fed ad lubitum, fasted 3 days, homozygous D298, homozygous N298 | {{230 Lkhagvadorj,S. 2009;}} |
| GSE13528-L | Liver | Fat | F | 6 months | fed ad lubitum, fasted 3 days, homozygous D298, homozygous N298 | {{230 Lkhagvadorj,S. 2009;}} |
| GSE14373 | Kidney | Fat | NA | NA | naïve, transplanted, non-transplanted, 4 or 24 hours, CO treated or untreated | {{292 Hanto,D.W. 2010;}} |
| GSE14643 | Heart | Muscle | F | 3 months | stem cell injection, saline injection | {{231 Jameel,M.N. 2010;}} |
| GSE14739-B | Fat | Fat | M/F | 80, 83, 87, 89 days | Male, Female, Large White, Iberian, Duroc, Youli | {{232 Perez-Enciso,M. 2009; 233 Yang,B. 2011;}} |
| GSE14739-G | Gonads | Reproduction | M/F | 80, 83, 87, 89 days | Male, Female, Large White, Iberian, Duroc, Youli | {{232 Perez-Enciso,M. 2009; 233 Yang,B. 2011;}} |
| GSE14739-H | Hypothalamus | Brain and Nervous System | M/F | 80, 83, 87, 89 days | Male, Female, Large White, Iberian, Duroc, Youli | {{232 Perez-Enciso,M. 2009; 233 Yang,B. 2011;}} |
| GSE14739-T | Thyroid Gland | Brain and Nervous System | M/F | 80, 83, 87, 89 days | Male, Female, Large White, Iberian, Duroc, Youli | {{232 Perez-Enciso,M. 2009; 233 Yang,B. 2011;}} |
| GSE14739-A | Adenohypophsis | Brain and Nervous System | M/F | 80, 83, 87, 89 days | Male, Female, Large White, Iberian, Duroc, Youli | {{232 Perez-Enciso,M. 2009; 233 Yang,B. 2011;}} |
| GSE14758 | Mediastinal Lymph nodes | Immune | NA | 7 days | Inoculated with PCV2, uninoculated, 1, 2, 5, 8, or 29 dpi | {{234 Tomas,A. 2010;}} |
| GSE14790 | Blood | Immune | NA | 7-36 days | Inoculated with PCV2, uninoculated, 0, 7, 14, 21 or 29 dpi | {{234 Tomas,A. 2010;}} |
| GSE15211 | Aortic Valve | Muscle | F | Adult | normotensive, hypertensive | {{293 Warnock,J.N. 2011;}} |
| GSE15256 | Ileum | Gut | NA | 5, 28 and 56 days | isolation/antibiotics/indoors, with sow indoors, outdoors, 5, 28 or 56 days | {{235 Mulder,I.E. 2009;}} |
| GSE16348 | Skeletal Muscle | Muscle | F | NA | sepsis, corticosteroids, NBMA/CS/sepsis, control | {{236 Banduseela,V.C. 2009;}} |
| GSE16855 | Neuroblasts/epithelial cells | Brain and Nervous System | F | neuroblasts from female newborn | neuroblasts, epithelial cells | {{237 Uebing-Czipura,A.U. 2009;}} |
| GSE17309 | Liver | Fat | M/F | 211 days | Male, Female, high, low feeding level | {{312 FernÃ¡ndez, AI. 2009;}} |
| GSE17492 | Spleen cells | Immune | M | Adult | Brucella suis infected, uninfected | {{238 Galindo,R.C. 2010;}} |
| GSE18343 | Endometrium | Embryo and Placenta | F | day 13 and 15 of psuedopregnancy | estrogen treated, non estrogen treated, day 13, day 15 | {{294 Ashworth,M.D. 2012;}} |
| GSE18359-A | Adipose Tissue | Fat | F | 6 Months | Low, High RFI, ad lib, calorie restricted | {{239 Lkhagvadorj,S. 2010;}} |
| GSE18359-L | Liver | Fat | F | 6 Months | Low, High RFI, ad lib, calorie restricted, | {{239 Lkhagvadorj,S. 2010;}} |
| GSE18467 | Cortical | Embryo and Placenta | NA | 60 or 80 days | day 60, day 80 | {{295 Nielsen,K.B. 2010;}} |
| GSE18641 | Endometrium | Embryo and Placenta | F | day 14 after insemination | Non-pregnant, pregnant, Yorkshire, Landrace | {{240 Ostrup,E. 2010;}} |
| GSE18653 | Longissimus Dorsi | Muscle | M | 6 or 7 months | Korean Native Pig, Yorkshire, 6 or 7 months | {{241 Kim,S.S. 2009;}} |
| GSE19275 | Gluteus Medius Muscle | Muscle | M | 190 days | High, low carcass/plasma/muscle fat content | {{242 Canovas,A. 2010;}} |
| GSE21096 | Heart | Muscle | F | NA | control, RCO, SWOP, RCS | {{243 Depre,C. 2010;}} |

^a^ Experiment ID: Gene Expression Omnibus GEO Series identifier

^b^ NA: not available, M/F: male and female, M: male, F: female.
