# Supplementary material for: First survey and functional annotation of prohormone and convertase genes in the pig
Source: BMC Genomics. 2012 Nov 15;13:582. doi: 10.1186/1471-2164-13-582 (PMC3499383; doi:10.1186/1471-2164-13-582)
Supplement: Additional file 3 — Table S3. Statistical significance P-value corresponding to the comparison between groups within experiment. [file 1471-2164-13-582-S3.doc]

**Supplementary materials Table S3.** Statistical significance P-value corresponding to the comparison between groups within experiment.

|  |  |  |  | Immune^b^ |  |  |  |  | Emb. |  |  |  |  | CNS |  |  |  | Repro. |  |  |  |  | Muscle |  |  |  |  |  |  | Fat |  |  |  |  | Gut |  |
| --- | --- | --- | --- | --- | --- | --- | --- | --- | --- | --- | --- | --- | --- | --- | --- | --- | --- | --- | --- | --- | --- | --- | --- | --- | --- | --- | --- | --- | --- | --- | --- | --- | --- | --- | --- | --- |
| Symbol | Affy Probe^a^ | GSE73132 | GSE7314 | GSE11787 | GSE17492 | GSE14758-D | GSE14790 | GSE18467 | GSE18641 | GSE18343 | GSE11853 | GSE12705 | GSE16855 | GSE12604 | GSE14739-H | GSE14739-T | GSE14739-A | GSE11590 | GSE14739-G | GSE18653 | GSE19275 | GSE8974 | GSE14643 | GSE15211 | GSE21096 | GSE16348-D | GSE17309 | GSE14373 | GSE14739-B | GSE9333 | GSE18359-A | GSE18359-L | GSE13528-F | GSE13528-L | GSE13457 | GSE15256 |
| *ADM5* | Ssc.26627.1.A1_at | 5.40E-01 | 2.60E-02 | 8.00E-02 | 7.20E-02 | 8.10E-01 | 3.10E-01 | 1.10E-02 | 1.50E-01 | 3.70E-01 | 7.00E-01 | 1.80E-01 | 3.40E-01 | 5.30E-01 | 9.70E-01 | 1.90E-03 | 5.00E-01 | 3.90E-01 | 6.20E-02 | 8.10E-01 | 1.70E-02 | 1.20E-01 | 9.80E-01 | 1.30E-01 | 1.10E-01 | 2.50E-01 | 4.60E-01 | 9.90E-01 | 7.00E-02 | 4.80E-01 | 4.00E-01 | 3.10E-01 | 2.00E-01 | 3.30E-01 | 3.90E-01 | 4.90E-02 |
| *ADML* | Ssc.314.1.S1_at | 1.90E-02 | 3.40E-04 | 2.40E-02 | 5.20E-01 | 1.70E-03 | 7.50E-02 | 8.50E-01 | 8.60E-02 | 7.20E-01 | 4.70E-02 | 1.20E-02 | 2.20E-06 | 2.60E-01 | 5.20E-01 | 7.20E-01 | 8.70E-02 | 1.60E-01 | 5.20E-02 | 6.60E-01 | 2.90E-02 | 6.30E-04 | 2.80E-01 | 7.50E-01 | 2.20E-01 | 3.30E-01 | 6.00E-01 | 3.40E-01 | 7.60E-01 | 8.60E-01 | 2.00E-01 | 3.40E-01 | 1.60E-01 | 2.60E-01 | 3.00E-01 | 4.40E-04 |
| *ANF* | Ssc.16245.1.S1_at | 9.70E-01 | 5.00E-01 | 1.70E-01 | 5.90E-01 | 5.00E-01 | 2.30E-02 | 1.80E-01 | 7.80E-01 | 2.70E-01 | 7.90E-01 | 7.40E-02 | 3.50E-01 | 9.70E-01 | 9.30E-01 | 3.30E-01 | 5.40E-01 | 2.00E-01 | 8.60E-01 | 4.60E-01 | 2.50E-01 | 9.80E-01 | 1.20E-01 | 3.50E-01 | 2.30E-04 | 5.20E-02 | 6.30E-01 | 6.20E-01 | 1.50E-01 | 7.10E-01 | 2.20E-01 | 6.60E-01 | 4.20E-01 | 4.50E-01 | 7.80E-02 | 5.50E-01 |
| *ANFB* | Ssc.629.1.S1_at | 5.20E-01 | 3.70E-01 | 6.70E-01 | 9.60E-02 | 9.40E-01 | 2.10E-01 | 4.00E-01 | 3.00E-01 | 5.40E-01 | 1.10E-01 | 3.00E-01 | 1.90E-01 | 6.20E-01 | 7.30E-01 | 5.10E-01 | 1.00E-01 | 6.00E-02 | 6.60E-01 | 6.10E-01 | 8.20E-02 | 2.40E-02 | 1.20E-01 | 3.80E-01 | 1.10E-03 | 9.60E-02 | 3.10E-01 | 3.60E-01 | 2.30E-01 | 1.80E-01 | 3.60E-01 | 9.40E-01 | 6.40E-01 | 9.20E-01 | 6.00E-01 | 5.50E-01 |
| *ANFC* | Ssc.23867.1.A1_at | 9.20E-01 | 1.30E-01 | 3.40E-01 | 7.40E-02 | 5.10E-01 | 3.20E-01 | 6.10E-01 | 4.70E-01 | 3.10E-01 | 5.90E-01 | 2.90E-05 | 9.40E-02 | 4.20E-01 | 9.40E-04 | 3.20E-01 | 4.80E-01 | 1.60E-01 | 6.10E-01 | 1.40E-01 | 2.40E-02 | 9.70E-01 | 9.40E-01 | 1.20E-01 | 1.00E-02 | 7.30E-01 | 5.90E-01 | 5.80E-01 | 7.70E-01 | 3.70E-01 | 6.50E-01 | 8.80E-01 | 8.20E-02 | 9.70E-01 | 5.90E-01 | 1.20E-01 |
| *AUGN* | Ssc.22487.1.S1_at | 4.10E-05 | 9.00E-03 | 3.90E-01 | 6.10E-01 | 1.90E-01 | 7.30E-04 | 9.00E-01 | 7.10E-01 | 6.50E-01 | 4.00E-01 | 1.50E-01 | 3.50E-01 | 1.30E-01 | 4.70E-02 | 2.80E-01 | 9.00E-02 | 1.20E-01 | 7.90E-02 | 6.00E-01 | 8.80E-01 | 3.90E-01 | 6.90E-01 | 2.90E-01 | 1.70E-04 | 7.20E-01 | 6.40E-01 | 3.80E-01 | 3.40E-01 | 9.10E-01 | 5.20E-01 | 1.20E-01 | 2.70E-04 | 3.60E-01 | 7.90E-01 | 3.40E-01 |
| *CART* | Ssc.15900.1.S1_at | 2.00E-01 | 8.40E-02 | 7.70E-01 | 4.60E-02 | 7.10E-01 | 1.40E-02 | 3.40E-01 | 3.90E-01 | 2.90E-01 | 3.40E-01 | 2.30E-03 | 3.60E-01 | 7.60E-01 | 3.60E-01 | 2.40E-03 | 2.50E-01 | 1.50E-01 | 3.20E-01 | 6.50E-01 | 4.60E-01 | 6.00E-01 | 4.30E-01 | 2.10E-02 | 1.00E-02 | 7.60E-02 | 4.60E-01 | 9.40E-01 | 1.60E-01 | 7.90E-01 | 7.60E-01 | 7.40E-01 | 1.70E-01 | 5.00E-01 | 5.90E-01 | 2.60E-01 |
| *CCKN* | Ssc.717.1.S1_at | 5.40E-01 | 6.00E-01 | 5.30E-01 | 5.00E-02 | 9.50E-01 | 1.20E-03 | 1.60E-01 | 9.80E-02 | 5.30E-01 | 5.30E-01 | 1.10E-01 | 6.30E-01 | 1.60E-01 | 2.60E-01 | 4.10E-01 | 5.00E-01 | 1.80E-01 | 1.20E-07 | 1.40E-01 | 2.80E-02 | 9.40E-01 | 5.80E-01 | 3.80E-01 | 2.10E-04 | 5.90E-02 | 5.20E-01 | 2.50E-01 | 8.20E-01 | 3.90E-01 | 5.30E-01 | 6.90E-01 | 1.80E-01 | 1.20E-02 | 2.30E-01 | 1.10E-01 |
| *CMGA* | Ssc.4653.1.S1_at | 1.20E-01 | 7.30E-01 | 5.00E-01 | 2.60E-01 | 9.70E-01 | 9.50E-02 | 1.40E-01 | 8.20E-01 | 4.10E-01 | 9.40E-01 | 2.00E-01 | 4.10E-01 | 2.70E-01 | 5.00E-01 | 8.70E-01 | 5.30E-01 | 1.50E-01 | 4.70E-01 | 1.00E+00 | 9.80E-02 | 4.30E-01 | 8.30E-01 | 8.40E-01 | 9.80E-02 | 1.00E-01 | 7.20E-01 | 7.40E-01 | 3.30E-01 | 3.30E-01 | 4.30E-01 | 7.80E-01 | 1.20E-03 | 3.30E-01 | 9.80E-01 | 7.60E-04 |
| *COLI* | Ssc.14556.1.S1_at | 5.90E-01 | 3.70E-01 | 6.00E-01 | 2.10E-01 | 6.50E-01 | 8.10E-02 | 2.40E-03 | 6.30E-01 | 9.20E-01 | 7.00E-01 | 1.20E-01 | 8.70E-01 | 6.40E-01 | 9.10E-01 | 3.50E-01 | 2.90E-01 | 1.50E-01 | 7.40E-01 | 1.10E-01 | 3.40E-01 | 8.20E-01 | 4.90E-01 | 1.60E-01 | 5.70E-02 | 1.00E-01 | 6.20E-01 | 9.50E-02 | 8.70E-01 | 3.60E-01 | 4.00E-01 | 5.70E-01 | 4.00E-01 | 9.20E-01 | 1.00E-01 | 2.00E-01 |
| *CRSP1* | Ssc.3741.1.S1_at | 9.00E-01 | 1.60E-01 | 8.10E-01 | 1.50E-02 | 2.50E-01 | 1.80E-01 | 4.40E-01 | 3.70E-01 | 7.70E-01 | 7.40E-01 | 7.10E-02 | 7.00E-02 | 4.80E-01 | 7.70E-01 | 6.50E-01 | 6.10E-03 | 1.40E-01 | 6.60E-01 | 7.90E-01 | 1.60E-01 | 2.60E-01 | 7.80E-01 | 2.80E-01 | 2.10E-02 | 4.50E-01 | 4.40E-01 | 1.10E-01 | 1.70E-01 | 4.40E-01 | 6.10E-01 | 5.50E-01 | 6.00E-01 | 3.30E-01 | 6.80E-01 | 4.40E-01 |
| *CRSP2* | Ssc.18558.1.S1_at | 8.10E-01 | 5.30E-01 | 6.70E-01 | 1.00E-01 | 5.10E-01 | 8.10E-03 | 2.20E-01 | 4.10E-01 | 8.80E-01 | 5.50E-01 | 3.30E-05 | 3.20E-01 | 5.40E-01 | 6.00E-01 | 1.70E-01 | 5.10E-01 | 1.40E-01 | 7.10E-01 | 1.60E-01 | 1.80E-02 | 7.00E-02 | 6.00E-01 | 4.80E-02 | 2.10E-02 | 7.80E-02 | 6.00E-01 | 6.10E-01 | 2.00E-01 | 8.00E-01 | 6.30E-01 | 9.90E-01 | 3.10E-01 | 9.10E-01 | 3.30E-02 | 7.10E-01 |
| *CRSP3* | Ssc.17879.1.S1_at | 9.30E-01 | 1.50E-01 | 9.20E-01 | 6.30E-02 | 6.50E-01 | 2.30E-03 | 1.10E-01 | 4.20E-01 | 8.50E-01 | 8.00E-01 | 1.20E-01 | 8.50E-01 | 8.80E-01 | 4.00E-01 | 7.30E-01 | 3.30E-02 | 1.60E-01 | 2.20E-01 | 8.10E-02 | 6.00E-02 | 4.80E-01 | 8.20E-01 | 1.60E-01 | 5.30E-02 | 6.50E-02 | 3.80E-01 | 9.00E-01 | 5.00E-02 | 5.10E-01 | 9.20E-01 | 9.90E-01 | 5.00E-01 | 8.60E-01 | 1.80E-01 | 4.50E-01 |
| *EDN1* | Ssc.9364.1.S1_at | 2.00E-03 | 5.20E-04 | 1.50E-01 | 1.20E-01 | 8.70E-02 | 6.60E-02 | 9.60E-01 | 1.20E-01 | 2.70E-01 | 1.50E-01 | 4.70E-01 | 9.70E-01 | 7.90E-01 | 7.10E-01 | 6.60E-01 | 5.40E-02 | 1.60E-01 | 1.60E-02 | 9.90E-01 | 1.00E-02 | 3.40E-01 | 4.30E-01 | 2.10E-01 | 3.20E-01 | 1.00E-01 | 5.80E-01 | 9.60E-01 | 9.90E-01 | 7.90E-01 | 6.80E-01 | 3.40E-01 | 3.70E-03 | 2.90E-01 | 2.80E-01 | 2.40E-01 |
| *GALA* | Ssc.713.1.S1_at | 3.80E-01 | 1.10E-01 | 8.30E-01 | 2.00E-02 | 4.00E-01 | 2.50E-04 | 1.30E-02 | 7.30E-01 | 7.50E-01 | 6.80E-01 | 1.10E-03 | 8.80E-01 | 5.10E-01 | 8.90E-01 | 3.30E-01 | 4.10E-01 | 1.50E-01 | 6.10E-01 | 1.00E-01 | 3.90E-02 | 9.40E-01 | 5.10E-01 | 2.70E-01 | 3.00E-02 | 1.10E-01 | 5.60E-01 | 6.70E-01 | 2.30E-01 | 7.60E-01 | 7.40E-01 | 9.80E-01 | 7.80E-01 | 9.50E-01 | 5.70E-01 | 9.40E-04 |
| *GALP* | Ssc.4875.1.S1_at | 1.10E-01 | 3.90E-01 | 1.90E-01 | 2.20E-01 | 5.20E-01 | 1.30E-03 | 2.90E-01 | 6.40E-01 | 4.60E-01 | 7.20E-01 | 2.10E-04 | 8.10E-01 | 4.70E-01 | 6.20E-01 | 1.50E-01 | 6.70E-02 | 1.40E-01 | 1.70E-02 | 1.90E-01 | 8.30E-04 | 5.50E-01 | 6.10E-01 | 4.90E-01 | 7.20E-03 | 3.80E-01 | 3.30E-01 | 7.20E-01 | 9.00E-02 | 6.10E-01 | 4.30E-01 | 6.60E-01 | 8.40E-01 | 7.90E-01 | 1.70E-01 | 1.50E-01 |
| *GAST* | Ssc.644.1.S1_at | 5.80E-01 | 3.20E-01 | 6.30E-01 | 1.40E-01 | 2.40E-01 | 4.60E-01 | 1.50E-01 | 2.60E-01 | 8.60E-01 | 5.90E-01 | 5.30E-04 | 1.90E-01 | 3.40E-01 | 9.00E-01 | 3.10E-01 | 5.30E-01 | 1.50E-01 | 5.20E-01 | 9.70E-01 | 3.70E-01 | 4.80E-01 | 9.00E-01 | 1.10E-01 | 6.80E-02 | 1.50E-01 | 7.40E-01 | 7.30E-01 | 6.60E-01 | 1.60E-01 | 5.10E-01 | 8.50E-01 | 2.30E-01 | 3.60E-01 | 5.30E-01 | 6.80E-01 |
| *GHRL* | Ssc.440.1.S1_at | 2.40E-01 | 1.70E-01 | 7.50E-01 | 1.40E-01 | 5.40E-01 | 5.80E-01 | 2.00E-01 | 6.70E-01 | 8.20E-01 | 7.30E-01 | 7.50E-01 | 2.90E-01 | 6.40E-01 | 9.80E-01 | 3.60E-01 | 2.50E-01 | 1.60E-01 | 5.50E-01 | 7.50E-01 | 1.30E-02 | 7.40E-01 | 4.90E-01 | 1.40E-01 | 3.40E-02 | 1.60E-01 | 8.20E-01 | 5.30E-01 | 5.40E-01 | 6.20E-01 | 9.30E-01 | 9.90E-01 | 3.30E-01 | 3.20E-01 | 3.30E-01 | 4.70E-02 |
| *GLUC* | Ssc.17225.1.S1_at | 4.40E-01 | 3.30E-01 | 9.30E-01 | 1.90E-01 | 4.60E-01 | 2.30E-02 | 2.60E-01 | 3.00E-01 | 6.10E-01 | 2.20E-01 | 5.30E-05 | 6.80E-01 | 8.00E-01 | 7.00E-01 | 5.00E-01 | 3.60E-01 | 5.60E-04 | 6.70E-01 | 7.90E-02 | 4.70E-01 | 7.50E-01 | 2.90E-01 | 3.50E-01 | 7.50E-03 | 1.20E-01 | 4.10E-01 | 4.60E-01 | 3.10E-01 | 4.90E-01 | 6.20E-01 | 8.60E-01 | 4.10E-01 | 9.10E-01 | 2.80E-01 | 3.50E-03 |
| *GON1* | Ssc.16310.1.S1_at | 9.70E-01 | 1.80E-01 | 9.10E-01 | 4.40E-02 | 6.10E-01 | 4.00E-05 | 2.20E-01 | 4.50E-01 | 7.80E-01 | 7.80E-01 | 2.10E-04 | 8.40E-01 | 6.90E-01 | 4.10E-01 | 3.20E-01 | 2.20E-01 | 1.40E-01 | 7.50E-01 | 3.20E-01 | 2.00E-02 | 8.30E-01 | 3.30E-01 | 3.10E-02 | 4.10E-02 | 5.80E-02 | 6.00E-01 | 5.20E-01 | 1.30E-01 | 5.70E-01 | 9.70E-01 | 9.10E-01 | 5.70E-01 | 5.20E-01 | 1.20E-01 | 4.00E-01 |
| *HEPC* | Ssc.376.1.S1_at | 9.10E-01 | 4.80E-01 | 9.40E-01 | 2.60E-02 | 7.90E-01 | 1.20E-02 | 1.50E-01 | 9.50E-01 | 6.00E-01 | 6.70E-01 | 2.30E-01 | 5.50E-01 | 6.50E-01 | 8.20E-01 | 6.20E-01 | 1.50E-01 | 1.60E-01 | 8.40E-01 | 3.70E-01 | 2.90E-02 | 9.30E-01 | 2.10E-01 | 2.70E-01 | 3.10E-02 | 1.30E-01 | 1.50E-01 | 7.80E-01 | 8.30E-02 | 1.00E+00 | 4.50E-01 | 8.90E-02 | 3.30E-01 | 3.30E-01 | 3.40E-01 | 2.90E-01 |
| *IAPP* | Ssc.8324.1.A1_at | 4.40E-01 | 4.20E-01 | 7.20E-01 | 2.10E-02 | 7.50E-01 | 1.80E-01 | 1.30E-01 | 9.00E-01 | 5.70E-01 | 7.90E-01 | 1.20E-03 | 9.20E-01 | 9.70E-01 | 2.30E-01 | 3.50E-01 | 8.20E-02 | 1.20E-01 | 6.10E-01 | 1.20E-01 | 5.10E-02 | 7.70E-01 | 2.00E-01 | 8.00E-01 | 5.20E-02 | 5.80E-02 | 6.10E-01 | 6.80E-01 | 1.00E-01 | 5.30E-01 | 9.10E-01 | 9.10E-01 | 5.10E-01 | 7.60E-01 | 6.60E-01 | 2.20E-01 |
| *IGF1* | Ssc.16231.1.S1_a_at | 5.70E-01 | 4.30E-03 | 4.30E-01 | 1.10E-01 | 4.20E-01 | 1.50E-01 | 2.40E-01 | 3.70E-01 | 1.60E-01 | 9.50E-02 | 1.10E-02 | 4.70E-06 | 4.60E-01 | 7.60E-01 | 3.80E-01 | 1.60E-01 | 1.70E-01 | 8.60E-03 | 3.00E-01 | 5.30E-01 | 1.30E-01 | 5.70E-01 | 9.30E-01 | 2.20E-02 | 5.20E-02 | 7.40E-02 | 5.30E-01 | 4.40E-01 | 6.50E-01 | 5.60E-03 | 7.10E-03 | 6.10E-03 | 2.70E-01 | 4.30E-01 | 3.50E-01 |
|  | Ssc.16231.2.A1_a_at | 6.40E-01 | 1.20E-01 | 5.70E-01 | 2.20E-01 | 7.50E-01 | 1.20E-01 | 1.20E-01 | 3.70E-01 | 9.50E-01 | 7.80E-01 | 1.30E-02 | 7.70E-01 | 7.30E-01 | 8.40E-01 | 1.30E-01 | 4.70E-01 | 1.40E-01 | 7.90E-01 | 4.80E-01 | 1.70E-01 | 6.20E-01 | 2.30E-01 | 1.40E-01 | 6.00E-02 | 3.10E-01 | 6.70E-01 | 9.10E-01 | 3.80E-01 | 9.70E-01 | 5.10E-01 | 8.30E-01 | 2.10E-01 | 9.80E-01 | 5.80E-01 | 7.00E-01 |
|  | Ssc.16231.3.S1_a_at | 6.50E-01 | 8.10E-02 | 3.00E-01 | 1.40E-01 | 1.20E-01 | 1.00E-02 | 5.80E-01 | 3.80E-01 | 9.50E-02 | 5.30E-01 | 3.60E-02 | 2.40E-07 | 4.90E-01 | 9.70E-01 | 4.90E-01 | 5.40E-01 | 1.70E-01 | 7.20E-02 | 6.00E-01 | 7.10E-01 | 8.00E-02 | 1.00E+00 | 9.10E-01 | 2.10E-02 | 3.20E-01 | 1.50E-01 | 7.50E-01 | 2.80E-01 | 4.90E-01 | 3.50E-02 | 7.50E-02 | 1.30E-02 | 2.90E-01 | 1.10E-01 | 6.30E-02 |
| *IGF2* | Ssc.9365.1.S1_at | 3.90E-01 | 2.40E-01 | 2.70E-01 | 2.60E-01 | 9.40E-01 | 2.70E-03 | 2.00E-01 | 8.50E-01 | 6.20E-01 | 5.00E-01 | 8.90E-03 | 4.70E-01 | 4.30E-01 | 8.20E-01 | 1.50E-01 | 2.40E-01 | 7.80E-01 | 3.60E-01 | 4.10E-01 | 3.50E-01 | 6.60E-01 | 3.90E-01 | 3.10E-01 | 1.80E-02 | 2.70E-01 | 5.20E-01 | 8.20E-01 | 6.50E-01 | 7.80E-01 | 5.40E-01 | 7.80E-01 | 2.90E-01 | 8.70E-01 | 9.00E-01 | 1.70E-01 |
|  | Ssc.9365.2.S1_a_at | 3.40E-02 | 5.50E-02 | 1.00E-01 | 5.50E-03 | 2.70E-01 | 9.60E-04 | 8.60E-01 | 2.70E-03 | 7.10E-01 | 6.80E-01 | 2.60E-02 | 2.90E-01 | 3.20E-01 | 8.70E-02 | 4.40E-01 | 8.50E-01 | 1.40E-01 | 1.40E-01 | 5.10E-01 | 6.20E-02 | 1.10E-01 | 7.50E-01 | 6.30E-01 | 7.90E-02 | 4.20E-01 | 2.70E-01 | 1.80E-02 | 4.20E-03 | 6.20E-01 | 8.40E-01 | 8.40E-01 | 3.40E-02 | 1.80E-02 | 1.80E-01 | 2.50E-02 |
|  | Ssc.9365.3.S1_a_at | 1.90E-01 | 3.10E-03 | 1.50E-01 | 6.40E-02 | 1.80E-01 | 6.90E-03 | 9.40E-01 | 1.50E-02 | 5.90E-01 | 3.90E-01 | 3.50E-01 | 6.10E-02 | 5.70E-01 | 1.10E-02 | 6.30E-01 | 9.40E-01 | 1.70E-01 | 2.50E-01 | 7.30E-01 | 1.00E-01 | 9.50E-01 | 7.50E-01 | 3.60E-01 | 4.30E-02 | 1.60E-01 | 5.80E-01 | 5.00E-03 | 8.20E-02 | 5.90E-01 | 6.40E-01 | 9.00E-01 | 1.10E-01 | 3.80E-02 | 4.80E-01 | 2.80E-02 |
|  | Ssc.9365.3.S1_x_at | 3.90E-02 | 9.60E-02 | 2.00E-01 | 3.70E-02 | 2.60E-01 | 3.50E-02 | 7.20E-01 | 2.00E-02 | 7.50E-01 | 3.90E-01 | 1.90E-02 | 7.70E-01 | 6.00E-01 | 5.10E-01 | 5.50E-01 | 6.60E-01 | 1.50E-01 | 3.50E-01 | 5.60E-01 | 1.50E-01 | 2.00E-01 | 5.30E-01 | 4.10E-01 | 3.50E-02 | 2.50E-01 | 4.90E-01 | 9.30E-03 | 2.40E-02 | 5.70E-01 | 7.50E-01 | 7.80E-01 | 3.10E-02 | 2.60E-02 | 4.70E-02 | 6.50E-03 |
|  | Ssc.9365.4.S1_a_at | 7.50E-01 | 1.50E-01 | 7.20E-01 | 5.00E-01 | 6.00E-01 | 5.10E-03 | 2.40E-01 | 1.00E+00 | 9.30E-01 | 5.80E-01 | 5.20E-04 | 3.50E-01 | 4.70E-01 | 9.10E-01 | 2.00E-01 | 1.00E-01 | 1.60E-01 | 3.90E-01 | 5.70E-01 | 2.60E-02 | 8.50E-01 | 1.80E-01 | 9.90E-02 | 4.10E-02 | 2.60E-01 | 2.20E-01 | 8.90E-01 | 3.30E-01 | 7.30E-01 | 8.30E-01 | 9.10E-01 | 4.00E-01 | 2.30E-01 | 8.70E-02 | 5.00E-01 |
|  | Ssc.9365.5.A1_at | 6.90E-01 | 1.50E-01 | 2.70E-01 | 1.10E-01 | 3.30E-01 | 1.70E-03 | 8.50E-01 | 5.40E-01 | 8.00E-01 | 6.20E-01 | 1.90E-02 | 1.30E-01 | 3.40E-01 | 9.10E-01 | 7.90E-01 | 4.70E-01 | 1.60E-01 | 4.70E-01 | 4.10E-01 | 6.80E-02 | 8.90E-01 | 9.70E-01 | 1.60E-01 | 7.90E-02 | 2.50E-01 | 9.10E-01 | 9.70E-01 | 5.10E-02 | 4.70E-01 | 5.70E-01 | 8.10E-01 | 2.90E-01 | 9.40E-01 | 4.70E-02 | 4.40E-01 |
|  | Ssc.9365.5.S1_at | 9.30E-01 | 9.60E-01 | 8.20E-01 | 2.80E-02 | 2.30E-01 | 7.10E-04 | 1.80E-01 | 5.20E-01 | 2.80E-01 | 7.10E-01 | 5.30E-04 | 9.30E-01 | 4.80E-01 | 7.50E-01 | 5.20E-01 | 3.40E-01 | 1.50E-01 | 9.20E-01 | 5.70E-01 | 2.60E-01 | 4.10E-01 | 4.30E-01 | 1.20E-01 | 2.80E-02 | 3.10E-01 | 4.30E-01 | 3.40E-01 | 2.30E-01 | 4.50E-01 | 8.40E-01 | 6.20E-01 | 5.40E-01 | 5.30E-01 | 8.60E-02 | 3.60E-01 |
|  | Ssc.9365.5.S1_a_at | 6.30E-01 | 4.10E-01 | 1.00E-01 | 9.30E-02 | 1.20E-01 | 9.00E-03 | 8.70E-01 | 1.60E-02 | 2.10E-01 | 6.80E-01 | 3.00E-01 | 6.40E-02 | 6.00E-01 | 9.60E-03 | 6.30E-01 | 6.20E-01 | 1.70E-01 | 3.70E-01 | 3.80E-01 | 1.90E-01 | 8.40E-01 | 6.40E-01 | 7.30E-01 | 1.10E-01 | 3.80E-01 | 4.40E-01 | 3.70E-03 | 1.20E-01 | 6.50E-01 | 6.10E-01 | 9.30E-01 | 6.40E-02 | 3.70E-02 | 9.50E-02 | 2.00E-02 |
|  | Ssc.9365.6.A1_a_at | 1.90E-01 | 3.30E-01 | 5.30E-01 | 8.10E-01 | 4.20E-01 | 4.50E-02 | 2.30E-01 | 7.00E-01 | 9.10E-01 | 3.40E-01 | 7.70E-02 | 7.60E-01 | 6.50E-01 | 6.90E-01 | 6.10E-02 | 6.80E-02 | 1.60E-01 | 3.00E-01 | 2.60E-01 | 7.30E-01 | 7.60E-01 | 7.60E-01 | 7.00E-01 | 9.10E-02 | 3.40E-01 | 9.10E-01 | 9.20E-01 | 8.10E-01 | 6.00E-01 | 9.80E-01 | 7.60E-01 | 2.60E-01 | 9.30E-01 | 5.50E-02 | 7.10E-01 |
|  | Ssc.9365.6.A1_x_at | 4.20E-01 | 6.40E-01 | 9.90E-01 | 6.40E-01 | 6.10E-01 | 6.60E-03 | 3.40E-01 | 2.90E-01 | 3.00E-01 | 1.80E-01 | 6.20E-02 | 2.70E-01 | 7.50E-01 | 4.60E-01 | 6.00E-02 | 3.00E-01 | 5.30E-01 | 3.20E-01 | 2.60E-01 | 3.60E-01 | 7.30E-01 | 9.40E-01 | 5.30E-01 | 5.00E-03 | 1.70E-02 | 4.40E-01 | 4.60E-01 | 3.30E-01 | 2.80E-01 | 7.20E-01 | 9.90E-01 | 4.90E-01 | 6.80E-01 | 5.40E-01 | 2.80E-01 |
|  | Ssc.9365.6.S1_x_at | 3.20E-02 | 4.20E-02 | 1.10E-01 | 2.70E-02 | 1.10E-01 | 2.90E-03 | 1.30E-01 | 3.40E-02 | 7.00E-01 | 4.80E-01 | 1.20E-01 | 1.80E-02 | 3.40E-01 | 2.30E-03 | 5.70E-01 | 3.40E-01 | 1.40E-01 | 3.50E-01 | 3.30E-01 | 3.40E-01 | 7.30E-01 | 3.20E-01 | 2.40E-01 | 8.50E-03 | 1.10E-01 | 3.70E-01 | 2.00E-02 | 9.70E-02 | 9.20E-01 | 5.20E-01 | 7.80E-01 | 5.30E-02 | 5.30E-02 | 1.40E-01 | 5.30E-03 |
|  | Ssc.9365.7.A1_x_at | 7.00E-01 | 4.50E-01 | 3.30E-01 | 5.10E-01 | 6.70E-01 | 2.00E-01 | 1.90E-01 | 6.90E-01 | 5.30E-01 | 3.90E-01 | 2.60E-02 | 3.80E-01 | 4.50E-01 | 8.50E-01 | 1.80E-01 | 1.60E-01 | 2.20E-01 | 3.20E-01 | 4.50E-01 | 4.60E-01 | 8.70E-01 | 9.40E-01 | 8.10E-02 | 1.20E-01 | 3.30E-02 | 9.80E-01 | 9.30E-01 | 3.80E-01 | 4.90E-01 | 8.20E-01 | 8.40E-01 | 5.00E-01 | 9.90E-01 | 9.20E-02 | 1.90E-01 |
| *INS* | Ssc.583.1.S1_at | 8.50E-01 | 1.10E-01 | 7.70E-01 | 1.60E-02 | 6.20E-01 | 1.90E-02 | 2.20E-01 | 4.90E-01 | 8.90E-01 | 9.10E-01 | 4.40E-01 | 6.00E-01 | 4.00E-01 | 9.10E-01 | 2.20E-01 | 4.20E-01 | 1.50E-01 | 6.80E-01 | 3.80E-01 | 2.70E-02 | 8.60E-01 | 4.10E-01 | 1.00E+00 | 1.20E-02 | 1.00E-02 | 8.80E-01 | 6.30E-01 | 3.30E-02 | 9.10E-01 | 5.10E-01 | 9.80E-01 | 2.40E-01 | 7.60E-01 | 3.80E-02 | 1.00E-01 |
| *INSL3* | Ssc.11990.1.S1_at | 3.10E-01 | 8.90E-01 | 4.40E-01 | 6.30E-02 | 5.10E-01 | 7.20E-02 | 3.50E-01 | 6.10E-01 | 8.60E-01 | 8.80E-01 | 1.30E-03 | 2.00E-01 | 5.40E-01 | 8.10E-01 | 3.80E-01 | 4.70E-01 | 1.50E-01 | 7.20E-02 | 1.60E-01 | 2.60E-01 | 9.20E-01 | 3.50E-01 | 2.60E-01 | 7.90E-02 | 4.00E-01 | 7.50E-01 | 5.90E-01 | 8.00E-01 | 9.10E-01 | 5.30E-01 | 7.10E-01 | 6.80E-01 | 7.40E-01 | 8.10E-02 | 7.20E-01 |
| *MCH* | Ssc.3287.1.S1_at | 5.70E-02 | 1.90E-01 | 6.60E-01 | 2.60E-02 | 5.80E-01 | 4.80E-02 | 3.60E-01 | 9.20E-01 | 8.40E-01 | 8.50E-01 | 2.50E-01 | 9.40E-01 | 2.10E-01 | 3.50E-01 | 3.30E-01 | 1.60E-01 | 1.60E-01 | 6.10E-01 | 9.80E-01 | 1.80E-02 | 2.30E-01 | 8.80E-01 | 3.60E-01 | 7.80E-02 | 7.00E-02 | 4.90E-01 | 7.50E-01 | 1.10E-01 | 9.60E-01 | 8.10E-01 | 9.60E-01 | 4.70E-01 | 7.50E-01 | 6.10E-01 | 2.00E-01 |
| *MOTI* | Ssc.714.1.S1_at | 8.20E-01 | 1.80E-01 | 5.40E-01 | 1.90E-01 | 7.80E-01 | 2.20E-01 | 3.90E-01 | 9.60E-01 | 6.20E-01 | 6.10E-01 | 9.10E-01 | 7.90E-01 | 6.00E-01 | 8.20E-01 | 6.70E-01 | 1.10E-01 | 1.60E-01 | 7.30E-01 | 2.40E-01 | 1.00E-02 | 9.70E-01 | 2.70E-01 | 3.40E-01 | 3.80E-02 | 9.70E-02 | 4.80E-01 | 9.90E-01 | 5.60E-01 | 4.10E-01 | 6.40E-02 | 5.40E-01 | 2.30E-01 | 3.90E-01 | 6.60E-01 | 5.20E-01 |
| *NEU1* | Ssc.15668.1.A1_at | 5.60E-01 | 4.60E-02 | 5.40E-01 | 9.40E-02 | 2.80E-01 | 8.00E-03 | 5.50E-01 | 3.50E-01 | 6.30E-01 | 7.80E-01 | 6.30E-02 | 3.90E-01 | 6.30E-01 | 1.30E-01 | 3.90E-01 | 9.80E-01 | 1.40E-01 | 1.80E-01 | 1.20E-01 | 1.30E-02 | 7.40E-01 | 3.70E-01 | 5.10E-02 | 6.70E-03 | 4.80E-02 | 9.10E-01 | 5.50E-01 | 8.50E-02 | 4.60E-01 | 6.80E-01 | 9.60E-01 | 3.10E-01 | 7.60E-01 | 4.30E-02 | 2.40E-01 |
| *NEU2* | Ssc.4210.1.S1_at | 6.50E-01 | 3.30E-02 | 4.80E-01 | 2.00E-01 | 3.30E-01 | 6.90E-02 | 5.30E-01 | 5.30E-01 | 6.70E-01 | 4.70E-01 | 6.20E-01 | 1.20E-01 | 6.60E-01 | 1.60E-01 | 3.00E-01 | 7.20E-01 | 1.60E-01 | 4.30E-01 | 8.00E-02 | 3.50E-02 | 7.80E-01 | 4.60E-01 | 7.10E-02 | 2.90E-03 | 1.40E-02 | 7.30E-01 | 9.10E-01 | 1.50E-01 | 7.70E-01 | 3.20E-01 | 6.40E-01 | 4.60E-01 | 5.50E-01 | 4.20E-02 | 7.20E-01 |
| *NMB* | Ssc.2083.1.A1_at | 9.70E-01 | 3.80E-02 | 9.00E-01 | 2.20E-01 | 2.50E-01 | 3.20E-03 | 2.10E-01 | 1.40E-02 | 7.70E-01 | 8.40E-01 | 1.40E-02 | 5.00E-01 | 5.20E-01 | 2.10E-01 | 2.10E-01 | 1.50E-02 | 1.50E-01 | 6.80E-01 | 5.40E-01 | 2.10E-02 | 2.60E-01 | 6.20E-01 | 6.90E-02 | 4.40E-02 | 7.10E-02 | 4.70E-01 | 1.90E-01 | 1.20E-01 | 3.00E-01 | 1.80E-01 | 9.50E-01 | 6.50E-02 | 9.00E-01 | 1.20E-01 | 3.30E-01 |
| *NMU* | Ssc.12508.1.A1_at | 9.10E-01 | 4.80E-01 | 5.80E-01 | 1.50E-02 | 8.50E-01 | 1.40E-03 | 3.00E-01 | 1.30E-02 | 3.90E-01 | 8.50E-01 | 1.30E-01 | 6.90E-01 | 3.90E-02 | 5.50E-01 | 3.40E-01 | 1.70E-01 | 1.30E-01 | 5.60E-01 | 1.20E-01 | 1.30E-02 | 7.80E-01 | 2.00E-01 | 7.80E-01 | 5.00E-02 | 6.40E-02 | 8.40E-01 | 4.80E-01 | 7.20E-01 | 9.60E-01 | 5.80E-01 | 9.90E-01 | 7.60E-01 | 7.70E-01 | 7.40E-01 | 8.70E-01 |
| *NPW* | Ssc.15796.1.S1_at | 4.10E-01 | 2.80E-01 | 3.90E-01 | 3.20E-02 | 1.50E-01 | 6.20E-02 | 3.00E-01 | 5.00E-01 | 7.60E-01 | 4.90E-01 | 3.30E-05 | 6.60E-01 | 4.30E-01 | 9.40E-01 | 4.10E-01 | 5.20E-01 | 1.60E-01 | 6.30E-01 | 1.10E-01 | 2.20E-01 | 7.30E-01 | 8.50E-01 | 1.50E-01 | 3.90E-02 | 2.70E-01 | 8.40E-01 | 9.80E-01 | 6.70E-01 | 8.00E-01 | 6.80E-01 | 9.00E-01 | 2.80E-01 | 5.20E-01 | 5.60E-02 | 4.40E-01 |
| *NPY* | Ssc.15981.1.A1_at | 3.30E-01 | 2.40E-01 | 4.10E-01 | 1.80E-02 | 6.20E-01 | 6.40E-04 | 7.50E-01 | 9.30E-01 | 7.60E-01 | 8.80E-01 | 7.30E-04 | 7.60E-01 | 9.50E-01 | 8.50E-01 | 5.10E-01 | 1.00E-01 | 1.60E-01 | 5.50E-01 | 1.80E-01 | 1.90E-01 | 4.50E-01 | 5.80E-01 | 3.80E-01 | 3.00E-03 | 1.30E-01 | 6.30E-01 | 7.70E-01 | 2.30E-02 | 7.00E-01 | 5.00E-01 | 9.60E-01 | 2.10E-01 | 9.00E-01 | 6.20E-02 | 3.00E-01 |
|  | Ssc.15981.1.S1_at | 5.40E-01 | 8.30E-01 | 1.50E-01 | 2.20E-02 | 3.40E-01 | 1.30E-01 | 7.20E-02 | 1.40E-03 | 5.70E-01 | 9.70E-01 | 3.50E-04 | 8.10E-04 | 3.20E-01 | 9.80E-01 | 7.20E-01 | 2.30E-01 | 1.40E-01 | 7.30E-01 | 3.40E-01 | 2.90E-02 | 5.70E-01 | 4.40E-01 | 5.50E-01 | 3.40E-02 | 9.50E-02 | 5.00E-01 | 5.80E-01 | 8.40E-02 | 8.80E-01 | 7.30E-01 | 9.50E-01 | 5.10E-01 | 6.60E-01 | 7.20E-02 | 2.20E-01 |
| *OREX* | Ssc.15983.1.S1_at | 4.40E-01 | 6.70E-03 | 3.70E-01 | 1.10E-01 | 1.30E-01 | 4.10E-02 | 5.60E-01 | 2.20E-01 | 9.60E-01 | 7.50E-01 | 1.00E-01 | 5.10E-01 | 1.60E-01 | 2.00E-01 | 7.50E-01 | 6.30E-01 | 2.60E-01 | 8.40E-01 | 6.50E-01 | 1.70E-01 | 7.40E-01 | 2.80E-01 | 1.10E-01 | 9.90E-01 | 9.40E-02 | 8.30E-01 | 6.90E-01 | 2.00E-02 | 6.50E-01 | 4.10E-01 | 1.90E-01 | 2.70E-01 | 8.00E-01 | 5.70E-01 | 3.60E-01 |
| *PACA* | Ssc.27598.1.S1_at | 5.10E-01 | 4.00E-01 | 9.20E-01 | 2.70E-02 | 6.50E-01 | 2.60E-01 | 6.40E-01 | 5.80E-01 | 1.90E-01 | 5.30E-01 | 3.90E-04 | 1.10E-01 | 3.30E-01 | 2.30E-02 | 2.20E-01 | 7.40E-01 | 7.40E-01 | 8.00E-01 | 2.80E-01 | 2.30E-02 | 8.10E-01 | 2.00E-01 | 2.60E-01 | 2.30E-01 | 5.60E-01 | 6.00E-01 | 8.00E-01 | 5.60E-03 | 7.10E-01 | 4.00E-01 | 1.80E-01 | 4.40E-01 | 4.20E-01 | 9.30E-02 | 1.00E-01 |
| *PAHO* | Ssc.456.1.S1_at | 7.30E-01 | 1.50E-01 | 7.00E-01 | 3.00E-02 | 4.70E-01 | 9.40E-03 | 3.00E-01 | 4.20E-01 | 5.50E-01 | 8.20E-01 | 6.00E-05 | 6.10E-01 | 6.60E-01 | 9.80E-01 | 3.50E-01 | 3.00E-01 | 1.40E-01 | 5.80E-01 | 2.70E-01 | 3.10E-02 | 8.70E-01 | 3.10E-01 | 2.00E-01 | 3.20E-02 | 9.30E-02 | 4.90E-01 | 8.40E-01 | 1.60E-01 | 6.20E-01 | 8.60E-01 | 9.60E-01 | 4.50E-01 | 6.00E-01 | 1.70E-01 | 3.70E-01 |
| *PCSK1* | Ssc.17429.1.S1_at | 2.00E-01 | 2.80E-01 | 3.10E-01 | 8.90E-01 | 1.40E-01 | 1.70E-02 | 1.60E-01 | 4.90E-01 | 5.30E-01 | 4.20E-01 | 4.80E-03 | 7.50E-02 | 8.80E-01 | 9.90E-01 | 5.40E-01 | 9.80E-01 | 3.00E-01 | 3.70E-05 | 3.50E-01 | 9.30E-01 | 5.10E-02 | 1.80E-01 | 9.30E-02 | 9.40E-02 | 1.30E-01 | 4.60E-01 | 6.70E-01 | 6.20E-01 | 9.90E-01 | 7.50E-01 | 9.40E-01 | 2.50E-01 | 1.40E-01 | 8.10E-01 | 1.10E-02 |
| *PDGFA* | Ssc.6173.3.S1_a_at | 3.00E-01 | 5.20E-03 | 3.90E-01 | 2.20E-01 | 1.60E-01 | 1.30E-04 | 6.70E-02 | 4.50E-01 | 3.50E-01 | 3.50E-01 | 6.50E-03 | 1.20E-03 | 3.00E-01 | 9.90E-02 | 5.40E-01 | 1.90E-01 | 9.60E-02 | 1.10E-01 | 1.90E-01 | 7.70E-01 | 2.90E-01 | 2.60E-01 | 9.10E-01 | 1.40E-01 | 2.70E-02 | 1.10E-01 | 3.50E-01 | 6.10E-02 | 4.30E-01 | 4.50E-01 | 2.40E-01 | 1.00E+00 | 4.40E-01 | 6.90E-01 | 7.80E-07 |
| *PDYN* | Ssc.121.1.S1_at | 5.30E-01 | 4.30E-01 | 5.30E-01 | 1.10E-01 | 4.90E-01 | 2.10E-01 | 9.30E-01 | 9.90E-01 | 7.80E-01 | 6.20E-01 | 3.00E-03 | 4.90E-05 | 8.80E-01 | 3.50E-01 | 5.00E-01 | 5.10E-01 | 1.20E-01 | 7.20E-01 | 1.60E-01 | 4.60E-02 | 1.90E-01 | 2.50E-01 | 8.00E-01 | 2.50E-01 | 3.10E-02 | 5.00E-01 | 9.90E-01 | 5.10E-02 | 7.40E-01 | 4.60E-01 | 9.90E-01 | 6.20E-01 | 7.40E-01 | 3.40E-01 | 1.40E-01 |
| *PENK* | Ssc.11281.1.A1_at | 2.90E-02 | 1.20E-01 | 6.00E-01 | 1.60E-02 | 4.60E-01 | 5.80E-01 | 1.90E-01 | 5.40E-02 | 8.50E-02 | 7.90E-01 | 1.90E-04 | 9.30E-01 | 6.50E-01 | 4.50E-01 | 7.50E-01 | 5.00E-01 | 4.30E-01 | 2.00E-05 | 4.30E-02 | 3.50E-01 | 5.60E-01 | 2.70E-01 | 7.40E-01 | 6.20E-02 | 9.40E-01 | 5.60E-01 | 7.20E-01 | 6.90E-01 | 5.80E-01 | 7.60E-01 | 1.00E+00 | 5.80E-05 | 9.00E-01 | 2.80E-01 | 1.80E-08 |
|  | Ssc.11281.2.S1_at | 1.90E-01 | 4.30E-01 | 6.50E-01 | 9.50E-02 | 7.20E-01 | 2.60E-05 | 2.40E-01 | 9.40E-02 | 7.90E-01 | 6.30E-01 | 7.70E-06 | 2.40E-01 | 3.00E-01 | 6.80E-01 | 3.60E-01 | 4.60E-01 | 3.10E-01 | 7.20E-02 | 3.90E-01 | 8.60E-01 | 9.40E-01 | 3.90E-01 | 1.00E-01 | 1.90E-01 | 1.10E-01 | 6.10E-01 | 5.20E-01 | 4.50E-01 | 4.90E-01 | 3.60E-01 | 9.40E-01 | 6.40E-02 | 9.70E-01 | 3.40E-02 | 3.00E-01 |
| *PNOC* | Ssc.15910.1.A1_at | 9.00E-01 | 9.00E-01 | 9.00E-01 | 2.50E-01 | 5.00E-01 | 1.00E-01 | 1.60E-01 | 4.00E-01 | 5.10E-01 | 5.40E-01 | 6.80E-01 | 3.80E-01 | 5.20E-01 | 8.60E-01 | 7.00E-01 | 3.80E-01 | 1.60E-01 | 4.90E-01 | 6.30E-01 | 1.20E-01 | 8.70E-01 | 7.70E-01 | 9.70E-01 | 1.90E-01 | 3.50E-01 | 4.40E-01 | 1.80E-01 | 4.90E-01 | 4.90E-01 | 4.10E-01 | 2.10E-01 | 9.90E-02 | 2.40E-01 | 9.00E-01 | 5.00E-01 |
|  | Ssc.15910.1.S1_at | 2.90E-01 | 5.10E-01 | 4.60E-01 | 8.10E-02 | 6.00E-01 | 1.50E-01 | 5.70E-02 | 3.50E-01 | 8.90E-01 | 2.00E-01 | 1.20E-01 | 7.20E-01 | 7.90E-01 | 4.60E-01 | 5.00E-01 | 1.40E-01 | 1.60E-01 | 6.80E-01 | 4.10E-01 | 2.10E-02 | 9.90E-01 | 3.60E-01 | 5.40E-01 | 1.80E-01 | 1.90E-01 | 6.20E-01 | 7.40E-01 | 1.70E-01 | 5.70E-01 | 6.30E-01 | 8.60E-01 | 1.30E-01 | 7.80E-01 | 8.30E-01 | 1.60E-01 |
| *PTHR* | Ssc.9991.1.S1_at | 6.10E-02 | 4.20E-01 | 3.80E-01 | 8.70E-02 | 5.50E-01 | 3.40E-02 | 2.70E-01 | 7.60E-03 | 2.00E-01 | 1.50E-01 | 3.50E-07 | 2.60E-04 | 9.20E-02 | 1.80E-01 | 4.70E-01 | 1.80E-01 | 1.50E-03 | 7.10E-10 | 3.80E-02 | 1.80E-01 | 2.10E-01 | 3.80E-01 | 1.90E-01 | 3.20E-01 | 4.80E-01 | 9.90E-01 | 6.30E-01 | 4.50E-01 | 8.70E-01 | 4.30E-01 | 9.80E-01 | 3.20E-01 | 7.80E-01 | 3.60E-01 | 1.60E-01 |
| *PTHY* | Ssc.668.1.S1_at | 3.90E-02 | 3.20E-01 | 9.00E-01 | 1.30E-02 | 7.40E-01 | 8.10E-03 | 1.90E-01 | 5.10E-01 | 8.30E-01 | 6.80E-01 | 3.30E-04 | 5.60E-01 | 8.20E-01 | 5.30E-01 | 2.20E-01 | 6.10E-02 | 1.50E-01 | 5.70E-01 | 2.20E-01 | 7.90E-03 | 7.80E-01 | 6.10E-01 | 5.90E-01 | 2.50E-02 | 4.90E-02 | 6.20E-01 | 8.80E-01 | 1.20E-01 | 9.00E-01 | 7.60E-01 | 1.00E+00 | 5.90E-01 | 9.00E-01 | 9.40E-01 | 1.90E-01 |
| *REL1* | Ssc.162.1.S1_at | 6.50E-01 | 2.00E-02 | 8.90E-01 | 4.60E-02 | 9.80E-01 | 1.30E-03 | 3.40E-01 | 7.70E-01 | 4.50E-01 | 2.90E-01 | 4.50E-04 | 3.00E-01 | 8.90E-01 | 4.60E-01 | 2.80E-01 | 1.60E-01 | 2.40E-01 | 4.90E-01 | 4.70E-01 | 9.70E-03 | 9.50E-01 | 2.70E-01 | 4.90E-01 | 1.00E-01 | 1.40E-01 | 6.20E-01 | 6.10E-02 | 3.20E-02 | 5.10E-01 | 4.90E-01 | 1.00E+00 | 9.30E-01 | 1.00E+00 | 2.00E-01 | 8.50E-01 |
| *SCG1* | Ssc.15718.1.A1_at | 9.50E-01 | 3.10E-02 | 9.60E-01 | 8.20E-02 | 8.00E-01 | 1.90E-03 | 1.10E-01 | 8.10E-01 | 6.10E-01 | 6.60E-01 | 3.60E-05 | 8.30E-01 | 6.70E-01 | 5.20E-01 | 1.70E-01 | 9.80E-01 | 1.40E-01 | 7.80E-01 | 2.10E-01 | 3.90E-01 | 2.80E-01 | 3.10E-01 | 3.70E-01 | 2.40E-02 | 9.50E-02 | 7.10E-01 | 8.70E-01 | 6.90E-01 | 7.40E-01 | 3.80E-01 | 9.90E-01 | 4.90E-01 | 9.90E-01 | 9.00E-01 | 4.60E-09 |
| *SCG2* | Ssc.13645.1.A1_at | 8.40E-02 | 8.20E-03 | 6.90E-01 | 7.60E-02 | 3.90E-01 | 4.80E-05 | 3.20E-02 | 8.70E-01 | 8.30E-01 | 4.10E-01 | 1.70E-02 | 6.30E-01 | 6.70E-01 | 4.80E-01 | 1.80E-01 | 1.30E-01 | 1.60E-01 | 6.80E-01 | 1.50E-01 | 3.50E-01 | 2.40E-01 | 2.70E-01 | 3.90E-01 | 8.00E-03 | 1.60E-01 | 3.60E-03 | 8.40E-01 | 6.20E-01 | 8.40E-01 | 9.10E-01 | 9.50E-01 | 6.00E-01 | 8.30E-01 | 7.90E-01 | 6.60E-06 |
| *SCG3* | Ssc.6770.1.A1_at | 6.70E-01 | 2.60E-01 | 1.20E-03 | 7.50E-01 | 3.50E-02 | 7.70E-02 | 4.40E-01 | 5.10E-01 | 6.10E-01 | 7.30E-01 | 9.60E-05 | 8.70E-01 | 4.90E-01 | 5.80E-03 | 4.00E-01 | 3.80E-02 | 1.00E-03 | 4.60E-01 | 1.70E-01 | 1.00E-01 | 8.80E-01 | 2.90E-01 | 4.50E-01 | 6.20E-03 | 1.10E-01 | 6.40E-01 | 3.50E-01 | 4.50E-01 | 2.90E-01 | 5.70E-01 | 9.70E-01 | 6.20E-01 | 6.20E-01 | 4.10E-01 | 1.20E-01 |
| *SECR* | Ssc.710.1.S1_at | 3.90E-01 | 2.40E-02 | 1.00E+00 | 5.10E-02 | 4.40E-01 | 5.20E-02 | 5.90E-01 | 8.60E-01 | 8.20E-01 | 6.40E-01 | 3.50E-05 | 6.30E-01 | 1.90E-01 | 9.00E-02 | 2.90E-01 | 4.10E-01 | 9.30E-01 | 5.50E-01 | 4.70E-01 | 3.70E-01 | 2.60E-01 | 6.40E-01 | 1.70E-01 | 4.20E-02 | 8.00E-01 | 6.70E-01 | 2.10E-02 | 2.80E-01 | 5.30E-01 | 2.10E-01 | 3.90E-02 | 4.30E-01 | 6.40E-02 | 4.80E-01 | 1.20E-01 |
| *SMS* | Ssc.19520.1.A1_at | 7.60E-01 | 1.60E-01 | 9.40E-01 | 2.00E-01 | 1.00E+00 | 7.40E-05 | 4.80E-01 | 3.90E-02 | 1.30E-01 | 8.60E-01 | 6.80E-04 | 5.00E-01 | 7.20E-01 | 6.70E-01 | 3.00E-01 | 1.60E-01 | 2.40E-01 | 6.90E-01 | 4.70E-01 | 8.80E-01 | 2.50E-02 | 2.10E-01 | 3.30E-01 | 4.90E-02 | 2.80E-03 | 9.80E-01 | 1.70E-01 | 1.00E-01 | 7.60E-01 | 7.80E-01 | 9.80E-01 | 5.50E-01 | 4.40E-01 | 7.30E-01 | 2.90E-01 |
| *TKN1* | Ssc.18075.1.A1_at | 7.00E-01 | 7.10E-02 | 5.30E-01 | 1.50E-02 | 4.00E-01 | 8.00E-03 | 6.00E-01 | 3.00E-01 | 3.50E-01 | 5.40E-01 | 1.50E-01 | 7.30E-01 | 1.60E-01 | 9.30E-01 | 5.60E-01 | 1.80E-01 | 1.30E-01 | 6.60E-01 | 2.90E-01 | 6.30E-03 | 9.00E-01 | 1.60E-01 | 6.60E-01 | 7.20E-02 | 2.70E-02 | 7.50E-01 | 6.50E-01 | 7.70E-01 | 6.00E-01 | 5.40E-02 | 9.30E-01 | 5.20E-01 | 8.20E-01 | 5.40E-01 | 5.20E-01 |
|  | Ssc.18075.2.S1_at | 6.80E-01 | 8.40E-01 | 5.10E-01 | 1.00E-01 | 6.70E-01 | 6.30E-03 | 6.30E-01 | 4.30E-01 | 7.00E-01 | 7.20E-01 | 1.30E-04 | 6.00E-01 | 7.30E-01 | 8.90E-01 | 8.20E-01 | 2.00E-02 | 1.50E-01 | 6.50E-01 | 2.30E-01 | 9.10E-03 | 6.90E-01 | 5.50E-01 | 1.00E-01 | 2.50E-02 | 4.40E-02 | 6.40E-01 | 9.20E-01 | 2.20E-01 | 7.90E-01 | 7.50E-01 | 9.70E-01 | 1.20E-01 | 9.40E-01 | 5.20E-02 | 5.10E-01 |
| *TKN4* | Ssc.23153.1.S1_at | 7.20E-01 | 4.80E-01 | 5.00E-01 | 7.40E-02 | 7.10E-01 | 6.90E-02 | 2.00E-01 | 9.30E-01 | 1.00E+00 | 2.30E-01 | 5.60E-03 | 9.20E-01 | 2.90E-01 | 8.00E-01 | 4.30E-01 | 2.70E-01 | 1.30E-01 | 5.10E-01 | 3.60E-01 | 4.80E-02 | 4.40E-01 | 9.40E-01 | 5.50E-01 | 5.70E-01 | 1.00E-01 | 9.40E-01 | 7.60E-01 | 5.00E-01 | 6.10E-01 | 9.00E-01 | 8.80E-01 | 4.10E-01 | 1.90E-01 | 7.70E-01 | 7.00E-02 |
| *TKNK* | Ssc.19565.1.S1_at | 7.80E-01 | 5.10E-02 | 3.20E-01 | 2.10E-02 | 5.60E-02 | 1.90E-01 | 2.60E-01 | 6.10E-01 | 5.20E-01 | 5.30E-01 | 6.60E-01 | 8.00E-01 | 5.40E-01 | 8.10E-01 | 4.80E-01 | 2.50E-01 | 1.50E-01 | 2.50E-02 | 1.60E-01 | 1.20E-02 | 7.50E-01 | 5.50E-01 | 2.10E-01 | 1.40E-02 | 9.70E-02 | 5.50E-01 | 6.30E-01 | 4.20E-01 | 7.30E-01 | 5.20E-01 | 9.90E-01 | 6.60E-01 | 9.70E-01 | 8.70E-01 | 6.20E-01 |
|  | Ssc.19565.2.A1_at | 1.50E-01 | 1.90E-01 | 3.80E-01 | 5.40E-02 | 3.20E-02 | 5.30E-01 | 2.20E-01 | 3.20E-01 | 5.30E-01 | 7.10E-01 | 3.20E-01 | 1.90E-01 | 3.00E-01 | 7.00E-01 | 8.00E-01 | 3.30E-01 | 3.30E-01 | 1.40E-02 | 8.20E-01 | 3.50E-01 | 8.10E-01 | 2.30E-01 | 2.30E-01 | 4.50E-02 | 6.00E-02 | 6.30E-01 | 1.00E+00 | 6.30E-01 | 5.30E-01 | 2.30E-01 | 8.30E-01 | 3.10E-01 | 3.30E-01 | 6.80E-01 | 1.80E-01 |
| *UTS2* | Ssc.437.1.S1_a_at | 9.30E-01 | 3.90E-01 | 4.10E-01 | 2.00E-02 | 8.60E-01 | 3.40E-02 | 1.50E-01 | 6.60E-01 | 4.90E-01 | 7.60E-01 | 7.80E-05 | 4.70E-01 | 4.40E-01 | 8.90E-01 | 4.00E-01 | 1.20E-01 | 8.90E-02 | 5.50E-01 | 3.00E-01 | 1.10E-02 | 8.60E-01 | 3.60E-01 | 3.70E-01 | 3.50E-02 | 5.60E-02 | 8.20E-01 | 9.10E-01 | 4.60E-02 | 4.60E-01 | 8.70E-01 | 9.40E-01 | 8.30E-01 | 9.40E-01 | 1.40E-01 | 4.60E-01 |
| *VEGFC* | Ssc.12790.1.A1_at | 4.10E-01 | 2.30E-02 | 1.40E-02 | 7.10E-01 | 1.30E-01 | 1.20E-03 | 1.90E-02 | 7.80E-04 | 9.00E-01 | 8.70E-01 | 6.40E-01 | 1.50E-09 | 5.30E-01 | 5.50E-02 | 3.80E-01 | 1.50E-01 | 1.50E-01 | 4.40E-01 | 8.90E-01 | 1.90E-01 | 1.20E-01 | 9.50E-01 | 7.40E-03 | 4.30E-06 | 6.10E-02 | 6.70E-01 | 4.40E-01 | 5.70E-01 | 8.20E-01 | 6.20E-03 | 1.50E-01 | 5.30E-02 | 3.80E-01 | 5.10E-01 | 3.70E-03 |
| *VEGFD* | Ssc.29289.1.A1_at | 2.70E-01 | 2.30E-01 | 7.10E-01 | 1.20E-01 | 4.00E-01 | 1.00E-04 | 7.40E-02 | 1.40E-01 | 3.10E-01 | 2.70E-01 | 3.60E-05 | 3.70E-01 | 3.90E-01 | 3.00E-01 | 7.80E-01 | 4.60E-01 | 5.90E-01 | 7.80E-02 | 5.60E-01 | 5.30E-02 | 4.40E-01 | 4.60E-01 | 4.20E-01 | 2.90E-02 | 1.80E-01 | 6.10E-01 | 8.70E-01 | 6.50E-01 | 7.00E-01 | 1.10E-02 | 5.70E-01 | 2.10E-01 | 4.40E-01 | 6.40E-02 | 6.70E-01 |
| Prohormone Convertase |  |  |  |  |  |  |  |  |  |  |  |  |  |  |  |  |  |  |  |  |  |  |  |  |  |  |  |  |  |  |  |  |  |  |  |  |
| *PCSK1* | Ssc.141.1.S1_at | 9.50E-01 | 1.70E-01 | 8.60E-01 | 1.50E-02 | 5.80E-01 | 3.40E-04 | 2.70E-02 | 6.60E-01 | 5.10E-01 | 8.40E-01 | 1.40E-04 | 6.60E-01 | 5.00E-01 | 6.20E-02 | 9.10E-02 | 9.90E-03 | 6.50E-01 | 2.50E-01 | 1.50E-01 | 1.30E-02 | 8.50E-01 | 2.20E-01 | 2.80E-01 | 7.90E-02 | 3.40E-02 | 3.10E-01 | 7.80E-01 | 4.70E-01 | 2.80E-01 | 4.10E-01 | 1.00E+00 | 1.20E-01 | 8.40E-01 | 3.40E-01 | 6.50E-07 |
| *PCSK2* | Ssc.109.1.S1_at | 4.40E-01 | 2.70E-01 | 3.50E-01 | 2.50E-02 | 5.80E-01 | 6.50E-03 | 9.90E-02 | 4.20E-01 | 5.70E-01 | 8.30E-01 | 5.10E-01 | 4.20E-01 | 3.60E-01 | 4.60E-01 | 6.30E-01 | 5.10E-01 | 1.00E-01 | 5.70E-01 | 2.10E-01 | 1.40E-01 | 8.40E-01 | 1.90E-01 | 2.90E-01 | 1.30E-02 | 8.80E-02 | 3.10E-01 | 6.50E-01 | 2.50E-01 | 6.30E-01 | 4.30E-01 | 3.30E-01 | 5.10E-01 | 8.40E-01 | 7.40E-01 | 5.40E-01 |
| *PCSK7* | Ssc.5628.1.S1_at | 3.80E-01 | 2.70E-01 | 7.30E-01 | 2.90E-02 | 7.40E-02 | 1.30E-03 | 6.20E-01 | 2.40E-01 | 4.10E-01 | 7.30E-01 | 9.70E-04 | 1.00E-02 | 6.90E-01 | 1.30E-01 | 9.90E-01 | 5.30E-01 | 2.00E-01 | 1.10E-01 | 1.60E-01 | 1.30E-01 | 1.90E-01 | 2.40E-01 | 1.00E-01 | 2.40E-02 | 3.30E-01 | 4.30E-01 | 1.00E-01 | 5.90E-01 | 4.90E-01 | 9.20E-01 | 4.40E-01 | 5.70E-01 | 5.40E-01 | 8.40E-01 | 1.20E-03 |

^a^Affymetrix microarray gene probe identifier.

^b^ Experiment classes: Imm: primary immune-response tissues, Emb: embryo and placenta, CNS: brain and central nervous system, Repro: reproduction.
