# Supplementary material for: First survey and functional annotation of prohormone and convertase genes in the pig
Source: BMC Genomics. 2012 Nov 15;13:582. doi: 10.1186/1471-2164-13-582 (PMC3499383; doi:10.1186/1471-2164-13-582)
Supplement: Additional file 4 — Table S1. Main features of the 35 microarray experiments analyzed. [file 1471-2164-13-582-S4.doc]

**Supplementary materials Table S1.** Prohormone and convertase genes identified across multiple pig genome resources.

| Type^a^ | Symbol | Name | Genome Sequence^b^ | UniGene Identifier | UniProt Identifier | UniProt Evidence | UniProt SequenceStatus | Gene Database Identifier | Ensembl Gene Identifier | Ensembl DNA Identifier | Ensembl Protein Identifier |
| --- | --- | --- | --- | --- | --- | --- | --- | --- | --- | --- | --- |
| P | *ADM2* | *Intermedin* | complete | Not Found | F1RXU1 | Predicted | Fragment | 100517471 | ENSSSCG00000023749 | ENSSSCT00000022401 | ENSSSCP00000020970 |
| P | *ADM5* | *Adrenomedullin-5* | complete | Ssc.26627 | A5LHG2 | Protein | Complete | 100101476 | ENSSSCG00000003190 | ENSSSCT00000003545 | ENSSSCP00000003462 |
| P | *ADML* | *Adrenomedullin* | complete | Ssc.314 | P53366 | Protein | Complete | 397195 | ENSSSCG00000013408 | ENSSSCT00000014648 | ENSSSCP00000014253 |
| P | *ANF* | *Atrial natriuretic factor* | complete | Ssc.16245 | P24259 | Protein | Fragment | 397496 | ENSSSCG00000003430 | ENSSSCT00000003808 | ENSSSCP00000003721 |
| P | *ANFB* | *Natriuretic peptide B* | complete | Ssc.629 | P07634 | Protein | Complete | 396844 | ENSSSCG00000003431 | ENSSSCT00000003809 | ENSSSCP00000003722 |
| P | *ANFC* | *C-type natriuretic peptide* | complete | Ssc.23867 | P18104 | Protein | Complete | 493772 | ENSSSCG00000026852 | ENSSSCT00000028969 | ENSSSCP00000022217 |
| P | *APEL* | *Apelin* | complete | CU928865 | Not Found | Not Found | Not Found | 100625006 | Not Found | Not Found | Not Found |
| P | *AUGN* | *Augurin (ECRG4)* | complete | Ssc.22487 | F1SU23 | Predicted | Complete | 100512958 | ENSSSCG00000008142 | ENSSSCT00000008919 | ENSSSCP00000008693 |
| P | *CALC* | *Calcitonin/calcitonin gene-related peptide 1* | complete | Ssc.14052 | A6P7L6 | Protein | Complete | 100125547 | ENSSSCG00000026512 | ENSSSCT00000028268 | ENSSSCP00000022117 |
| P | *CALCalt* | *Preprocalcitonin gene-related peptide* | complete | Ssc.56129 | A6P7L7 | Protein | Complete | 100124407 | ENSSSCG00000026512 | ENSSSCT00000026203 | ENSSSCP00000027470 |
| P | *CART* | *Cocaine- and amphetamine-regulated transcript protein* | complete | Ssc.15900 | Q307W6 | Protein | Complete | 397252 | ENSSSCG00000016972 | ENSSSCT00000018482 | ENSSSCP00000017982 |
| P | *CCKN* | *Cholecystokinin* | complete | Ssc.717 | P01356 | Protein | Complete | 397468 | ENSSSCG00000011277 | ENSSSCT00000012346 | ENSSSCP00000012023 |
| P | *CMGA* | *Chromogranin-A* | complete | Ssc.4653 | P04404 | Protein | Complete | 397540 | ENSSSCG00000002456 | ENSSSCT00000002726 | ENSSSCP00000002657 |
| P | *COLI* | *Pro-opiomelanocortin* | complete | Ssc.14556 | P01192 | Protein | Complete | 396863 | ENSSSCG00000021155 | ENSSSCT00000032500 | ENSSSCP00000024582 |
| P | *CORT* | *Cortistatin* | complete | Not Found | F1RIF7 | Predicted | Fragment | 100526112 | ENSSSCG00000027822 | ENSSSCT00000003775 | ENSSSCP00000003688 |
| P | *CRF* | *Corticoliberin* | complete | Ssc.69887 | P06296 | Protein | Complete | 100127468 | ENSSSCG00000006215 | ENSSSCT00000006811 | ENSSSCP00000006626 |
| P | *CRSP1* | *CRSP1_PIG* | complete | Ssc.3741 | Q862B1 | Protein | Complete | 396563 | Not Found | Not Found | Not Found |
| P | *CRSP2* | *CRSP2_PIG* | complete | Ssc.18558 | Q766Y7 | Transcript | Complete | 396574 | ENSSSCG00000013387 | ENSSSCT00000014623 | ENSSSCP00000014228 |
| P | *CRSP3* | *CRSP3_PIG* | complete | Ssc.17879 | Q766Y6 | Transcript | Complete | 396573 | ENSSSCG00000013386 | ENSSSCT00000014621 | ENSSSCP00000014226 |
| P | *CRSP3alt* | *Calcitonin-2* | complete | Not Found | A0A761 | Transcript | Complete | Not Found | Not Found | Not Found | Not Found |
| P | *EDN1* | *Endothelin-1* | complete | Ssc.9364 | P09558 | Protein | Complete | 396915 | ENSSSCG00000001050 | ENSSSCT00000001144 | ENSSSCP00000001121 |
| P | *EDN2* | *Endothelin-2* | complete | Not Found | Not Found | Not Found | Not Found | Not Found | Not Found | Not Found | Not Found |
| P | *EDN3* | *Endothelin-3* | complete | Ssc.31972 | A5A752 | Transcript | Complete | 100049663 | ENSSSCG00000007527 | ENSSSCT00000008255 | ENSSSCP00000008036 |
| P | *GALA* | *Galanin* | complete | Ssc.713 | P07480 | Protein | Complete | 397465 | ENSSSCG00000012883 | ENSSSCT00000014084 | ENSSSCP00000013698 |
| P | *GALP* | *Galanin-like peptide* | complete | Ssc.4875 | Q9TT95 | Protein | Complete | 396772 | ENSSSCG00000003318 | ENSSSCT00000003683 | ENSSSCP00000003598 |
| P | *GAST* | *Gastrin* | complete | Ssc.644 | P01351 | Protein | Complete | 445524 | ENSSSCG00000021910 | ENSSSCT00000025368 | ENSSSCP00000022312 |
| P | *GHRL* | *Obestatin* | complete | Ssc.440 | Q9GKY5 | Transcript | Complete | 396728 | ENSSSCG00000011568 | ENSSSCT00000012660 | ENSSSCP00000012327 |
| P | *GIP* | *Gastric inhibitory polypeptide* | complete | Ssc.38713 | P01281 | Protein | Complete | 100621117 | ENSSSCG00000026330 | ENSSSCT00000030856 | ENSSSCP00000023744 |
| P | *GLUC* | *Glucagon* | complete | Ssc.17225 | P01274 | Protein | Complete | 397595 | ENSSSCG00000015895 | ENSSSCT00000017307 | ENSSSCP00000016844 |
| P | *GON1* | *Progonadoliberin-1* | complete | Ssc.16310 | P49921 | Protein | Complete | 397516 | ENSSSCG00000009651 | ENSSSCT00000010581 | ENSSSCP00000010305 |
| P | *GON2* | *Progonadoliberin-2* | Not found | Not Found | F1S8B1 | Predicted | Complete | 100523475 | Not Found | Not Found | Not Found |
| P | *GRP* | *Gastrin-releasing peptide* | complete | Ssc.13923 | P63153 | Protein | Fragment | Not Found | ENSSSCG00000004913 | ENSSSCT00000005421 | ENSSSCP00000005286 |
| P | *HEPC* | *Hepcidin* | complete | Ssc.376 | Q8MJ80 | Transcript | Complete | 397207 | ENSSSCG00000002886 | ENSSSCT00000003189 | ENSSSCP00000003108 |
| P | *IAPP* | *Islet amyloid polypeptide* | complete | Ssc.8324 | Q29119 | Transcript | Fragment | 100520838 | ENSSSCG00000000582 | ENSSSCT00000000626 | ENSSSCP00000000612 |
| P | *IGF1* | *Insulin-like growth factor 1* | complete | Ssc.16231 | P16545 | Transcript | Complete | 397491 | ENSSSCG00000000857 | ENSSSCT00000000936 | ENSSSCP00000000916 |
| P | *IGF2* | *Insulin-like growth factor 2* | fragment | Ssc.9365 | P23695 | Protein | Complete | 396916 | Not Found | Not Found | Not Found |
| P | *INS* | *Insulin* | complete | Ssc.583 | P01315 | Protein | Complete | 397415 | ENSSSCG00000021171 | ENSSSCT00000031129 | ENSSSCP00000025428 |
| P | *INSL3* | *Insulin-like 3* | complete | Ssc.11990 | P51461 | Transcript | Complete | 397024 | ENSSSCG00000013887 | ENSSSCT00000015173 | ENSSSCP00000014767 |
| P | *INSL5* | *Insulin-like 5* | complete | Not Found | Not Found | Not Found | Not Found | 100620109 | ENSSSCG00000028935 | ENSSSCT00000024209 | ENSSSCP00000024615 |
| P | *INSL6* | *Insulin-like 6* | complete | Ssc.46919 | F1SK47 | Predicted | Fragment | 100158105 | ENSSSCG00000005214 | ENSSSCT00000005747 | ENSSSCP00000005605 |
| P | *KISS1* | *Metastasis-suppressor KiSS-1* | complete | Ssc.73565 | B5M447 | Transcript | Complete | 100145896 | ENSSSCG00000015280 | ENSSSCT00000016651 | ENSSSCP00000016205 |
| P | *MCH* | *Pro-melanin-concentrating hormone* | complete | Ssc.3287 | Q9TTS8 | Transcript | Fragment | 396962 | ENSSSCG00000000858 | ENSSSCT00000000937 | ENSSSCP00000000917 |
| P | *MOTI* | *Motilin* | complete | Ssc.714 | P01307 | Protein | Complete | 397466 | ENSSSCG00000001521 | ENSSSCT00000001694 | ENSSSCP00000001650 |
| P | *NEU1* | *Oxytocin* | complete | Ssc.15668 | P01177 | Protein | Complete | 100152272 | ENSSSCG00000007164 | ENSSSCT00000007837 | ENSSSCP00000007626 |
| P | *NEU2* | *Neurophysin-II* | complete | Ssc.4210 | P01183 | Protein | Complete | 396995 | ENSSSCG00000007163 | ENSSSCT00000007836 | ENSSSCP00000007625 |
| P | *NEUT* | *Neurotensin* | complete | Ssc.38680 | F1SPX3 | Predicted | Complete | 100739079 | ENSSSCG00000000932 | ENSSSCT00000001018 | ENSSSCP00000000996 |
| P | *NMB* | *Neuromedin-B* | complete | Ssc.2083 | B0LUW4 | Protein | Complete | 100141313 | ENSSSCG00000025679 | ENSSSCT00000030819 | ENSSSCP00000027260 |
| P | *NMS* | *Neuromedin-S* | complete | Ssc.12508 | C3UZJ1 | Transcript | Fragment | 100294685 | Not Found | Not Found | Not Found |
| P | *NMU* | *Neuromedin-U* | complete | Ssc.12508 | P34964 | Protein | Fragment | 100523263 | ENSSSCG00000027954 | ENSSSCT00000027137 | ENSSSCP00000025674 |
| P | *NPB* | *Neuropeptide B* | complete | Ssc.82498 | Not Found | Not Found | Not Found | Not Found | Not Found | Not Found | Not Found |
| P | *NPFF* | *Neuropeptide FF* | complete | Ssc.44958 | F1SFP1 | Predicted | Complete | 100518250 | ENSSSCG00000000277 | ENSSSCT00000000298 | ENSSSCP00000000293 |
| P | *NPS* | *Neuropeptide S* | complete | Ssc.73596 | F1RSG4 | Predicted | Fragment | 100188981 | ENSSSCG00000010751 | ENSSSCT00000011763 | ENSSSCP00000011461 |
| P | *NPW* | *Neuropeptide W* | complete | Ssc.15796 | Q8MI35 | Protein | Complete | 396680 | ENSSSCG00000008037 | ENSSSCT00000008806 | ENSSSCP00000008582 |
| P | *NPY* | *Neuropeptide Y* | complete | Ssc.15981 | P01304 | Protein | Complete | 397304 | ENSSSCG00000016718 | ENSSSCT00000018199 | ENSSSCP00000017708 |
| P | *OREX* | *Orexin* | complete | Ssc.15983 | O77668 | Transcript | Complete | 397305 | ENSSSCG00000017410 | ENSSSCT00000018952 | ENSSSCP00000018447 |
| P | *OSTN* | *Osteocrin (Musclin)* | complete | Ssc.5148 | A5JHN9 | Transcript | Complete | 100049691 | ENSSSCG00000011815 | ENSSSCT00000012927 | ENSSSCP00000012586 |
| P | *OX26* | *Orexigenic neuropeptide QRFP* | complete | Not Found | F1S0X5 | Predicted | Complete | 100524361 | ENSSSCG00000005705 | ENSSSCT00000006274 | ENSSSCP00000006112 |
| P | *PACA* | *Pituitary adenylate cyclase-activating polypeptide* | complete | Ssc.27598 | P41535 | Protein | Complete | 414283 | ENSSSCG00000003698 | ENSSSCT00000004097 | ENSSSCP00000004005 |
| P | *PAHO* | *Pancreatic polypeptide* | complete | Ssc.456 | P01300 | Protein | Fragment | 397272 | ENSSSCG00000017368 | ENSSSCT00000018907 | ENSSSCP00000018403 |
| P | *PCSK1N* | *Proprotein convertase subtilisin/kexin type 1 inhibitor* | complete | Ssc.17429 | Not Found | Not Found | Not Found | 100621697 | ENSSSCG00000021328 | ENSSSCT00000025387 | ENSSSCP00000022400 |
| P | *PDGFA* | *Platelet-derived growth factor alpha polypeptide* | complete | Ssc.6173 | F1RIZ0 | Predicted | Complete | 100519764 | ENSSSCG00000007541 | ENSSSCT00000008273 | ENSSSCP00000008054 |
| P | *PDGFB* | *Platelet-derived growth factor beta polypeptide* | complete | Ssc.54182 | P20034 | Protein | Fragment | 100126843 | ENSSSCG00000021641 | ENSSSCT00000029224 | ENSSSCP00000024855 |
| P | *PDGFD* | *Platelet-derived growth factor D* | complete | Ssc.49835 | F1SV50 | Predicted | Fragment | 100524161 | ENSSSCG00000014994 | ENSSSCT00000016357 | ENSSSCP00000015918 |
| P | *PDYN* | *Proenkephalin-B* | complete | Ssc.121 | P01214 | Protein | Complete | 445529 | ENSSSCG00000007180 | ENSSSCT00000007854 | ENSSSCP00000007643 |
| P | *PENK* | *Proenkephalin* | complete | Ssc.11281 | Q7M3H2 / Q7M2Z7 | Protein | Fragment | 100152093 | ENSSSCG00000006243 | ENSSSCT00000006841 | ENSSSCP00000006655 |
| P | *PNOC* | *Prepronociceptin* | complete | Ssc.15910 | P55791 | Protein | Complete | 397257 | ENSSSCG00000009675 | ENSSSCT00000010609 | ENSSSCP00000010333 |
| P | *PROK2* | *Prokineticin 2* | fragment | EW633867 | Not Found | Not Found | Not Found | 100526076 | ENSSSCG00000026200 | ENSSSCT00000022311 | ENSSSCP00000023671 |
| P | *PRRP* | *Prolactin-releasing peptide* | fragment | Not Found | Not Found | Not Found | Not Found | Not Found | Not Found | Not Found | Not Found |
| P | *PTHR* | *Parathyroid hormone-related peptide* | complete | Ssc.9991 | Q866H2 | Transcript | Complete | 396951 | ENSSSCG00000000544 | ENSSSCT00000000587 | ENSSSCP00000000574 |
| P | *PTHY* | *Parathyroid hormone* | complete | Ssc.668 | P01269 | Protein | Complete | 399502 | ENSSSCG00000013394 | ENSSSCT00000014631 | ENSSSCP00000014236 |
| P | *PYY* | *Peptide YY* | complete | Ssc.63650 | P68005 | Protein | Fragment | 445018 | ENSSSCG00000025414 | ENSSSCT00000030078 | ENSSSCP00000023751 |
| P | *REL1* | *Pro-relaxin 1* | complete | Ssc.162 | P01348 | Protein | Complete | 396891 | ENSSSCG00000005216 | ENSSSCT00000005749 | ENSSSCP00000005607 |
| P | *REL3* | *Relaxin 3* | complete | Ssc.42647 | Q8HY17 | Protein | Complete | 503836 | ENSSSCG00000013765 | ENSSSCT00000015039 | ENSSSCP00000014634 |
| P | *RES18* | *Regulated endocrine-specific protein 18* | complete | Ssc.49266 | F1SR77 | Predicted | Complete | 100154377 | ENSSSCG00000016219 | ENSSSCT00000017660 | ENSSSCP00000017182 |
| P | *RFRP* | *Neuropeptide VF precursor* | complete | Ssc.75350 | C4P9W1 | Transcript | Fragment | 100302024 | ENSSSCG00000016712 | ENSSSCT00000018193 | ENSSSCP00000017702 |
| P | *SCG1* | *Secretogranin-1* | complete | Ssc.15718 | Q9GLG4 | Protein | Complete | 397154 | ENSSSCG00000007045 | ENSSSCT00000007714 | ENSSSCP00000007507 |
| P | *SCG2* | *Secretogranin-2* | complete | Ssc.13645 | Q5FZP5 | Transcript | Complete | 497237 | ENSSSCG00000023032 | ENSSSCT00000024662 | ENSSSCP00000027906 |
| P | *SCG3* | *Secretogranin-3* | complete | Ssc.6770 | F1RYP7 | Predicted | Complete | 100154760 | ENSSSCG00000004630 | ENSSSCT00000005113 | ENSSSCP00000004989 |
| P | *SECR* | *Secretin* | complete | Ssc.710 | P63298 | Protein | Complete | 397464 | ENSSSCG00000012852 | ENSSSCT00000014047 | ENSSSCP00000013662 |
| P | *SLIB* | *Somatoliberin* | complete | Ssc.71374 | P01287 | Protein | Complete | 100499556 | ENSSSCG00000007332 | ENSSSCT00000008023 | ENSSSCP00000007809 |
| P | *SMS* | *Somatostatin* | complete | Ssc.19520 | P01168 | Protein | Complete | 494469 | ENSSSCG00000011808 | ENSSSCT00000012920 | ENSSSCP00000012579 |
| P | *SPXN* | *Spexin* | complete | Ssc.57764 | F1SR03 | Predicted | Fragment | 100155886 | ENSSSCG00000000578 | ENSSSCT00000000622 | ENSSSCP00000000608 |
| P | *TIP39* | *Parathyroid hormone 2* | complete | Not Found | F1RHZ | Predicted | Complete | 100515141 | ENSSSCG00000003171 | ENSSSCT00000003521 | ENSSSCP00000003438 |
| P | *TKN1* | *Tachykinin, precursor 1* | complete | Ssc.18075 | F1SF85 | Predicted | Complete | 100525179 | ENSSSCG00000023592 | ENSSSCT00000027581 | ENSSSCP00000020239 |
| P | *TKN4* | *Tachykinin-4* | complete | Ssc.23153 | F1RTB7 | Predicted | Complete | 100511101 | ENSSSCG00000017554 | ENSSSCT00000019109 | ENSSSCP00000018603 |
| P | *TKNK* | *Tachykinin 3* | complete | Ssc.19565 | P67934 | Protein | Complete | 492314 | ENSSSCG00000000418 | ENSSSCT00000000452 | ENSSSCP00000000446 |
| P | *TOR2X* | *Torsin family 2, member A* | fragment | Ssc.67158 | B6VD08 | Transcript | Fragment | 100519815 | ENSSSCG00000021924 | ENSSSCT00000026282 | ENSSSCP00000024349 |
| P | *TRH* | *Prothyroliberin* | complete | Not Found | P62968 | Protein | Fragment | 100513309 | ENSSSCG00000011596 | ENSSSCT00000012693 | ENSSSCP00000012360 |
| P | *UCN1* | *Urocortin* | Not found | Not Found | F8R6K7 | Predicted | Complete | Not Found | Not Found | Not Found | Not Found |
| P | *UCN2* | *Urocortin 2* | complete | Not Found | F1SKM2 | Predicted | Complete | 100521865 | Not Found | Not Found | Not Found |
| P | *UCN3* | *Urocortin 3* | complete | Not Found | F1RYW0 | Predicted | Complete | 100737810 | ENSSSCG00000011143 | ENSSSCT00000012195 | ENSSSCP00000011882 |
| P | *UTS2* | *Urotensin 2* | complete | Ssc.437 | Q95J46 | Transcript | Complete | 397268 | ENSSSCG00000023079 | ENSSSCT00000026053 | ENSSSCP00000019973 |
| P | *UTS2B* | *Urotensin II-related peptide* | complete | Not Found | F1SFH3 | Predicted | Fragment | 100626084 | ENSSSCG00000011816 | ENSSSCT00000012928 | ENSSSCP00000012587 |
| P | *VEGFC* | *Vascular endothelial growth factor C* | complete | Ssc.12790 | F1RT19 | Predicted | Fragment | 100525960 | ENSSSCG00000015770 | ENSSSCT00000017171 | ENSSSCP00000016710 |
| P | *VEGFD* | *Vascular endothelial growth factor D* | complete | Ssc.29289 | F1SQU4 | Predicted | Complete | 100155670 | ENSSSCG00000012135 | ENSSSCT00000013274 | ENSSSCP00000012918 |
| P | *VGF* | *Neurosecretory protein VGF* | fragment | Ssc.90772 | Not Found | Not Found | Not Found | 100624333 | ENSSSCG00000022576 | ENSSSCT00000024207 | ENSSSCP00000024335 |
| P | *VIP* | *Vasoactive intestinal peptide* | complete | Ssc.47759 | E0Y441 | Protein | Complete | 100500718 | ENSSSCG00000004078 | ENSSSCT00000004507 | ENSSSCP00000004405 |
| C | *7B2* | *Neuroendocrine protein 7B2* | complete | Ssc.155 | P01165 | Protein | Complete | 397110 | ENSSSCG00000004807 | ENSSSCT00000005306 | ENSSSCP00000005176 |
| C | *FURIN* | *Furin* | complete | Ssc.94009 | F1RMJ1 | Predicted | Complete | 100156882 | ENSSSCG00000001817 | ENSSSCT00000002036 | ENSSSCP00000001987 |
| C | *PCSK1* | *Proprotein convertase subtilisin/kexin type 1 PC1/3* | complete | Ssc.92884 | Q28959 | Transcript | Complete | 397103 | ENSSSCG00000014169 | ENSSSCT00000015478 | ENSSSCP00000015068 |
| C | *PCSK2* | *Neuroendocrine convertase 2* | complete | Ssc.109 | Q03333 | Transcript | Complete | 445533 | ENSSSCG00000007083 | ENSSSCT00000007755 | ENSSSCP00000007546 |
| C | *PCSK4* | *Proprotein convertase subtilisin/kexin type 4* | complete | Ssc.47037 | Not Found | Not Found | Not Found | 100626523 | ENSSSCG00000024379 | ENSSSCT00000028148 | ENSSSCP00000027290 |
| C | *PCSK5* | *Proprotein convertase subtilisin/kexin type 5* | incomplete | Ssc.43614 | Not Found | Not Found | Not Found | 100519237 | ENSSSCG00000023520 | ENSSSCT00000024731 | ENSSSCP00000019835 |
| C | *PCSK6* | *Proprotein convertase subtilisin/kexin type 6* | incomplete | Ssc.73551 | F1RZ92 | Predicted | Fragment | 100152144 | ENSSSCG00000005785 | ENSSSCT00000006361 | ENSSSCP00000006199 |
| C | *PCSK7* | *Proprotein convertase subtilisin/kexin type 7* | complete | Ssc.5628 | F1SJT0 | Predicted | Complete | 100523009 | ENSSSCG00000015072 | ENSSSCT00000016438 | ENSSSCP00000015996 |
| C | *PCSK9* | *Proprotein convertase subtilisin/kexin type 9* | complete | Ssc.84357 | Not Found | Not Found | Not Found | 100620501 | ENSSSCG00000025020 | ENSSSCT00000029382 | ENSSSCP00000023105 |

^a^ P: prohormone gene, C: prohormone convertase gene.

^b^ Genome sequence found: complete or incomplete in the pig genome assembly, found in the Traces archive, or not found in any genome repository.
